# Characterization of gliadin, secalin and hordein fractions using analytical techniques

**DOI:** 10.1038/s41598-021-02099-0

**Published:** 2021-11-30

**Authors:** Monika Rani, Dalbir Singh Sogi, Balmeet Singh Gill

**Affiliations:** Department of Food Science and Technology, Guru Nanak Dev University, Amritsar, Punjab India

**Keywords:** Biochemistry, Molecular biology, Plant sciences

## Abstract

Prolamins, alcohol soluble storage proteins of the *Triticeae* tribe of Gramineae family, are known as gliadin, secalin and hordein in wheat, rye and barley respectively. Prolamins were extracted from fifteen cultivars using DuPont protocol to study their physiochemical, morphological and structural characteristics. SDS-PAGE of prolamins showed well resolved low molecular weight proteins with significant amount of albumin and globulin as cross-contaminant. The β-sheet (32.72–37.41%) and β-turn (30.36–37.91%) were found higher in gliadins, while α-helix (20.32–28.95%) and random coil (9.05–10.28%) in hordeins. The high colloidal stability as depicted by zeta-potential was observed in gliadins (23.5–27.0 mV) followed secalins (11.2–16.6 mV) and hordeins (4.1–7.8 mV). Surface morphology by SEM illustrated the globular particle arrangement in gliadins, sheet like arrangement in secalins and stacked flaky particle arrangement in hordeins fraction. TEM studies showed that secalin and hordein fractions were globular in shape while gliadins in addition to globular structure also possessed rod-shaped particle arrangement. XRD pattern of prolamin fractions showed the ordered crystalline domain at 2θ values of 44.1°, 37.8° and 10.4°. The extracted prolamins fractions showed amorphous as well as crystalline structures as revealed by XRD and TEM analysis. Space saving hexagonal molecular symmetry was also observed in TEM molecular arrangement of prolamins which has profound application in development of plant-based polymers and fibres.

## Introduction

Prolamin is a group of heterogeneous mixture of alcohol soluble polypeptide (30–75 kDa MW) chains, which constituted the major storage protein of the *Triticeae* tribe, usually known as gliadins in wheat, secalin in rye and hordein in barley. The members of this tribe share closely resembled storage proteins. Prolamin is characterized by having high proportion of glutamine and proline and low proportion of arginine, lysine and histidine^1^. The gliadins, secalins and hordein are known to trigger/cause the celiac or coeliac disease (CD) in genetically susceptible individuals characterized by severe damage of jejunal mucosa (villi) by immune mediated reactions leading to chronical distension of abdomen, malnourishment, diarrhoea, stunted growth, depression, loss of appetite and weight^2^.

A recent meta-analysis revealed that the global CD pooled sero-prevalence was about 1.4%^2^. Previously, CD was considered as a Western or European disease but it has been reported from the different regions of the world such as Europe and Oceania (0.8%), Asia (0.6%), Africa and North America (0.5%) and South America (0.4%)^3^. Main factors for its dissemination are dietary, genetic^2^ and agricultural practices. Catassi et al.^4^ observed the high prevalence of this disease among Saharawi individuals (especially infants) and reported 55 individuals out of 989 were diagnosed with this disease. The probable reason of higher prevalence in African people was related to genetic, environmental as well abrupt change in diet from breast milk, camel milk, dates and sugar to wheat products especially bread as staple food^2^. In India, CD prevalence was more in wheat consuming states i.e. northern population (≈1.2%) than rice consuming states i.e. southern population (≈0.13%)^3^.

Prolamins are also gaining research interests as a potential plant-based source for hydrogels, films and nano-particle for controlled drugs and phyto-chemicals delivery^5–7^. Till date, only gliadin nano-particles have been successfully used for the delivery of drugs and nutrients^6–8^. Gliadin has also been used in development of plant-based biopolymers and fibres and has been reported to have high mechanical strength and hydrolytic stability compared to other biopolymers or fibres^9,10^. Numerous publications are available on gliadin^11–25^, while a scanty research has been conducted on secalin^13,16,26,27^and hordein^13,14,16,26,28–30^ especially on rye and barley cultivars for their molecular structure and chemical profile.

Field et al.^26^ studied the purification and characterization of secalin via electrophoresis, chromatography, amino acid composition/sequencing and compared it with the reported values of gliadin and hordein. The electro-spinnability of ethanol extracted gliadin and hordein in post spun fibre was also studied and compared using different analytical methods in the report of Wang and Chen^14^. Paananen et al.^31^ studied the gliadin protein interations using atomic force microscopy and postulated the rod shape of ω-gliadin, globular shape of α-gliadin and their hydrophobic intractions. Schalk et al.^13^ isolated, concentrated and purified the prolamin fractions, as a reference material and characterized each fraction with elctrophoretic, RP-HPLC, N-terminal sequence analysis and LC-ESI-QTOF-MS, using ethanol extraction procedure. Rasheed et al.^19^ studied the SE-HPLC, RP-HPLC, Small Angle X-ray Scattering, wide Angle X-ray Scattering and FTIR of modified gliadin and glutenin fractions by ethanol extraction protocol.

Different scientists have used different prolamin extraction methods and each protocol has its own drawbacks and benefits. The traditional ethanol extraction procedures not only extracts very small amount of ω-prolamin fractions but also exhibits cross-contamination with glutenin subunits (Siddiqi et al.^32^), while another method developed by Fu and Kovac^33^ and modified by DuPont et al.^11^, uses NaI in combination with small amount of propanol, extracted most of the prolamin fractions with neglible amount of cross contamination with glutenin subunits. The properties of the prolamin’s fraction obtained from different extraction method has been studied for gliadin^34–37^ but information on secalin and hordein could not be traced. Literature revealed that comparative analysis of gliadins, secalins and hordeins with any extraction techniques has not been carried out using Dynamic Light Scattering, Scanning/Transmission Electron Microscopy, Fourier-transform infrared spectroscopy and X-ray Diffraction techniques.

Comprehensive knowledge about the molecular, morphological and structural characteristics will help in better understanding the behaviour of prolamin extracted with DuPont et al.^11^ protocol in cereal crops. An attempt has been made to study the characteristics of gliadins, hordeins and secalins from DuPont et al.^11^ protocol for color (Hunter Color analysis), amino acid composition (Amino Acid Analyzer), secondary structure (Fourier-transform infrared spectroscopy), polypeptide profile (SDS-PAGE), hydrodynamic size (Dynamic Light Scattering), surface charge (Zeta Potential), morphology (Scanning Electron Microscopy and Transmission Electron Microscopy), elemental composition (Energy Dispersive X-Ray) and internal structure (Transmission Electron Microscopy and X-ray Diffraction).

## Materials and methods

### Materials

All the experimental work and collection of samples was done in accordance with the relevant national and international guidelines. The cereal grains of rye cultivars (MCTLG-1, MCTLG-2, MCTLG-3, MCTLG-4 and MCTLG-5) and wheat cultivars (HPW-42, HPW-147, HPW-155, HPW-236, HPW-249 and HPW-349) were obtained from agricultural university; Chaudhary Sarwan Kumar Himachal Pradesh Krishi Vishvavidyalaya (CSK HPKV), Palampur, India, which is located at latitude 32° 6′ 52″ N longitude 76° 33′ 24’’ E, and altitude 1614 m above sea level. The barley cultivars (BH-393, BH-902, BH-946 and BH-959) were obtained from Chaudhary Charan Singh Haryana Agricultural University (CCS HAU), Hisar, India latitude 29° 8′ 57.08″ N, longitude 75° 43′ 17.95″ E and altitude 212.78 m above sea level, Brabender Quadrumat Junior Mill (Brabender OHG, Duisburg, Germany) was used to mill the conditioned grains to obtained flour having extraction rate of 72%, 68% and 60% for wheat, rye, and barley respectively. It was stored at –20 °C and thaws before analysis (25 °C for 2 h). All the chemicals used were of analytical grade.

## Methods

### Prolamin extraction

Gliadins, secalins and hordeins were extracted from wheat, rye and barley respectively by following the procedure of DuPont et al.^11^. Briefly, 1 g of flour was taken in 10 mL extraction solution (0.3 M NaI solution containing 7.5% 1-propanol), shaken for 20 min and centrifuged (5810R, Eppendorf, Hamburg, Germany) for 10 min at 4500xg to collect the supernatant. Extraction process was repeated with the residues. The supernatants of both the fractions were pooled and transferred to round-bottom glass flask. 0.1 M ammonium acetate in methanol was added using a ratio of 4:1 :: ammonium acetate: supernatant, shaken thoroughly and kept at − 20 °C for 48 h to precipitate the prolamins. The solution was centrifuged at 3600×*g* (5810R, Eppendorf, Hamburg, Germany) to obtain the prolamin fraction as pellet. The precipitates were dissolved in 0.1 M acetic acid and also washed from the sides of the glass tube with 0.1 M acetic acid and freeze dried (HetoPowerDry, Allerod, Denmark). Freeze dried prolamin powders from wheat, rye and barley were termed as gliadins, secalins and hordeins fractions respectively. These fractions were stored in airtight plastic vials at -20 °C for further use.

### Protein determination

Protein content of freeze-dried powder of prolamin fractions was measured in triplicate by the Kjeldahl method (*N* × 5.7 for gliadin; *N* × *5.83* for secalin and hordein) following standard method of American Association of Cereal Chemists (46-12.01)^38^.

### Color of prolamin fraction

The color of freeze-dried powder of prolamin fractions was measured using Hunter Colour lab (Hunter Associates Laboratory Inc., Reston, USA)^39^. The instrument was calibrated with black and white standard tiles and then color of powdered sample was measured. The CIE values L*(0 darkness, 100 lightness), a* (+ red, –green color) and b* (+ yellow, –blue color) of the prolamin fractions were observed.

The hue angle (H^o^) is a color appearance parameter which refers as quantitative attribute of color lies between an angle of 0°–360° with 0° representing red, 90° for yellow, 180° for green and 270° for blue. While, Chroma refers to intensity of color purity or color strength. It is expressed as either grey or the pure hue (red/yellow/green/ blue etc.).

Hue and Chroma were calculated using the following Equations:

$${\text{Hue angle }}\left( {{\text{H}}^{{\text{o}}} } \right) = {\text{tan}}^{{ - 1}} \left( {{\text{b}}^{*} /{\text{a}}^{*} } \right)$$

$${\text{Chroma }}\left( {{\text{C}}^{*}} \right) = \left( {{\text{a}}^{{*2}} + {\text{b}}^{{*2}} } \right)^{{0.5}}$$

### SDS-PAGE of prolamin fractions

Sodium Dodecyl Sulphate–Polyacrylamide Gel Electrophoresis (SDS-PAGE) was used to analyse the extracted prolamin fractions by adopting the method of Siddiqi et al.^39^. 10 mg of prolamin fraction was thoroughly mixed with 1 ml of 2 × Laemmli sample buffer solution (pH 6.8 containing 62.5 mM Tris–HCl, 25% glycerol, 5% *ß*-mercaptoethanol, 2% SDS, 0.01% bromophenol blue) in 1.5 ml Eppendorf tubes. The tubes were vortexed to disperse the proteins and then shaken using an orbital shaker operated for 1 h at 151 rpm and 45 °C. Samples were heated at 100 °C for 5 min and then centrifuged (RC 4815S, Eltek, Mumbai, India) at 11000×*g* for 20 min.

SDS-PAGE of prolamin fraction was performed by loading 10 μl of prepared sample supernatant in each well and resolved in 12% resolving gel at a constant current of 25 mA (Mini-Protean Tetra Cell, Bio-Rad Laboratories, Hercules, USA).

When the tracking dye reached the bottom of the gel, plates were disassembled to remove the gel which was stained overnight in the staining solution (0.1% Coomassie Brilliant Blue-R250 in 40% methanol and 10% acetic acid). The gels were destained using 30% methanol and 10% acetic acid in deionized water.

The broad range molecular marker (GeNei, Bangalore, India) containing myosin (205 kDa), phosphorylase B (97.4 kDa), bovine serum albumin (66.0 kDa), ovalbumin (44.0 kDa), carbonic anhydrase (29.00 kDa), soyabean trypsin inhibitor (20.10 kDa), lysozyme (14.30 kDa), aprotinin (6.50 kDa) and insulin (3.50 kDa) was used for estimation of MW. The quantification of destained gel was carried out using Bio-Rad EZ Imager (Bio-Rad Laboratories, Hercules, USA).

Classification of total prolamin was done according to Schalk et al.^13^. The relative proportion of prolamin was calculated using band percentage corresponding to the total extractable proteins. SDS-PAGE gels were performed in duplicates. Bio Rad Image Lab software 6.1 was used as working station and the maximum background subtraction was performed to all lane.

### Amino acid analysis of prolamin fractions

Analysis of prolamin fraction was performed by following the procedure of Siddiqi et al.^39^ with slight modification**.** Briefly, 5 mg of prolamin were hydrolysed in clean dried screw capped glass test tubes which were priorly dipped whole night in 2 N HCl to avoid any sort of contamination. Hydrolysis was carried out using 6 N HCl containing 0.1% *ß*-mercaptoethanol in autoclave at 110 °C ± 2 °C for 16 min screw tight capped closure test tubes to minimize the loss of digestion solvent during hydrolysis process. The filtrate was evaporated in a small round bottomed rotary flask under vacuum at 40 °C to dryness in a rotary evaporator (Buchi, Fawil, Switzerland). A suitable volume of 0.1 N HCl was added to each dried film of the hydrolyzed sample to dissolve all the soluble materials and then filtered through 0.22 µm filter paper (Millipore, Merck Life Science Private Limited, Mumbai, India). Amino acid analysis was performed using a Nexera Amino acid Analyzer (Shimazdu, Kyoto, Japan) equipped with a pre-column derivatization using three derivatizing reagents such as mercaptopropionic acid, o-phthaladehyde and 9-fluorenylmethyl chloroformate. A C-18 column (Waters-Spherisorb ODS2 Column; 5 µm, 80 Å, 4.6 mm × 250 mm) having pH stability 2–8 was used for chromatographic separation. Analysis was performed using 20 mmol/L phosphate (potassium) buffer (pH 6.5) as solvent A and 45/40/15 acetonitrile/methanol/water as solvent B. The separation was obtained at a flow rate of 1 ml/min using a gradient elution that allowed 2% B at 0.01st min, followed by linear raise of eluent B to 50% at 41st min and then decreased to 2% B at 43rd min. The temperature of the column oven was set at 40 °C and injection volume to 1 μL. Resolution of amino acid derivatives was performed with the help of fluorescence detector having excitation and emission set at 330 nm and 450 nm respectively. Labsolutions LC/GC (Shimadzu, Kyoto, Japan) was used as a working station. The amino acid standard mixture was prepared by mixing eighteen amino acids (SRL, Mumbai, India) in 0.1 N HCl which included Aspartic acid (Asp), Glutamic acid (Glu), Serine (Ser), Glycine (Gly), threonine (Thr), Histidine (His), Alanine (Ala), Arginine (Arg), Tyrosine (Tyr), Valine (Val), Methionine (Met), Cystine (Cys), Phenylalanine (Phe), Tryptophan (Trp), Isoleucine (Ileu), Leucine (Leu), Lysine (Lys) and Proline (Pro). Each amino acid was identified by running it separately to determine its retention time. Then the mixture of 18 amino acids was named accordingly from the retention time of the individual amino acids as provided in Figure S2. The concentration of amino acids was estimated using single point calibration with no correction factor. The estimation of acid digested sample was carried out by comparing the area under the peak of standard mixture of each amino acid with that of the samples (Fig. 2 & Figure S3). Glutamine (Gln) and asparagine (Asn) were deaminated to glutamic acid (Glu) and aspartic acid (Asp) during acid hydrolysis^40^. Therefore, glutamic acid and aspartic acid were represented by combination of acid and its amide derivative as Glu + Gln and Asp and Asn.

### Fourier-transform infrared spectroscopy (FTIR)

FTIR spectral of prolamin fraction was recorded using FTIR/FIR Spectrometer (Perkin Elmer Inc, Waltham USA). The KBr pellet was made using finely grounded KBr salt (250 mg) with dried protein (concentration ≈1–5% Weight). The finely grounded mixture was then uniformly distributed in a suited cell and pressed with the force around 10 tons to yield fine transparent pellets (≈1–2 mm thickness) using hydraulic press. A pellet of pure KBr salt was used as a reference. Each sample was measured at least in triplicate, with total of 256 scans performed in the 400–4000 cm^−1^ region at 2 cm^−1^ resolutions. The spectral data acquisition and reference subtraction was performed using spectrum software, while the baseline correction, deconvolution of hidden peaks detection and curve fitting to obtain area under the peak were calculated using Origin Pro (version 2021, OriginLab Corporation, Northampton, USA).

### Peak fitting

The non-linear fitting of spectral 1600–1700 amide I region data was performed by adopting the procedure of Sadat & Joye^41^ using Peak analyzer-Levenberg–Marquardt algorithm to know the secondary structural components of the protein. Spectral 1600–1700 region plot was first subjected to baseline correction using second derivative (Zeros) method. Through peak analyzer, the hidden peaks were spotted on baseline corrected plot using second derivative method by setting the Savitsky-Golay function as smooth derivative and polynomial order as 2. After detecting the peak position based on negative peaks on secondary derivative, the multiple peak fitting was performed using the Voigt function (combination of Gaussian function and Lorentzian function). During initializing the parameters, fixed the y axis and perform simplex fit till the model (fitted cumulative curve) comes near to the observed (original curve) data. Then after iterations were performed till the model curve superimpose the observed curve and the best fit was achieved. Goodness-of-fit test was monitored to achieve best peak fitting i.e. chi square value (< 1E−6), R2 value (> 0.99), adjusted R2 value (> 0.99) and fit status. The relative area percentage of each structural components (α-helix, β-sheet, β-turn and random coil) was calculated by dividing the sum of area of assigned specific band position to that of total area.

### Dynamic light scattering (DLS) measurements

The hydrodynamic diameter (D_h_) and zeta potential (ζ-potential) values of proteins were monitored on Zetasizer Nano ZS (Malvern Instruments Ltd., Worcestershire, UK) equipped with a helium–neon (He–Ne) laser (632.8 nm, 4mW), at back scattering angle of 173° to the incident beam and operated at 25 °C. The instrument monitors, time-dependent fluctuation in the light scattered by molecules present in solution to determine the diffusion rate due to the Brownian motion at a fixed scattering angle. The extracted prolamin(s) (1 mg ml^−1^) were prepared in acetonitrile: water: formic acid (50:50:0.1) solution. The refractive index values of the solvent system acetonitrile: water: formic acid (50:50:0.1) solution has been taken from the literature^42^. To avoid the formation of protein aggregates, the samples were vortexed gently and then filtered through 0.22 µm Millipore filters prior to measurements. An average of three measurements for each sample was considered as an experimental data.

### Scanning electron microscopy (SEM) and energy dispersive X-ray analyser (EDX)

The morphology of the isolated freeze-dried prolamin powder was studied with Carl Zeiss Supra-55 SEM (Carl Zeiss, Oberkochen, Germany) at an accelerating voltage of 10–15 kV. The samples were mounted on stubs using double-sided adhesive tape. Prior to SEM imaging, all samples were sputter-coated with gold for 2 min by Quorum Sputtered Coater (Q150R ES, Laughton, East Sussex, UK). Further, the elemental composition was analysed with Energy dispersive X-ray spectrometer (Oxford Instruments Nano Analysis & Asylum Research, High Wycombe, UK).

### X-ray diffraction (XRD)

XRD studies, in the 2θ (Bragg’s angle) range of 10–60° were performed using X-ray diffractometer (Shimadzu 7000, Shimadzu Corporation, Kyoto, Japan) in Bragg–Brentano geometry with Cu K_α_ radiation (λ = 1.5405 A^o^). Some of the prolamins were found to be nano-crystalline in nature i.e. these are composed of crystallites or grains that are the ordered building blocks units repeated periodically and separated by disordered grain boundaries. Debye–Scherrer equation was used to calculate the particle crystallite size on corresponding diffraction pattern based on the dimension of full-width at half-maximum (FWHM) values.

$$D=\frac{K\lambda }{\beta .cos\theta }$$

where, D is the mean size of the ordered (crystalline) domains, λ is the X-ray wavelength in nanometre (nm), β is the peak width of the diffraction peak profile at half of the maximum height (FWHM) in radians, θ is the Bragg angle, which can be in degrees or radians, cos θ corresponds to the same number and *K* is a constant related to crystallite shape, normally it can be taken as 0.89 or 0.9 for Full Width Half Maximum (FWHM) of spherical crystals with cubic unit cells^43^**.**

Further, inter-planar spacing (d), was calculated from the peak positions using Bragg's equation

$$2\;{\text{d}}\;{\text{sin}}\theta = {\text{n}}\lambda$$

where d is inter-planar spacing, n is order of diffraction.

### Transmission electron microscopy (TEM)

The structures of different Prolamin fractions were investigated using Transmission Electron Microscope (JEM-2100, JEOL TEM, Tokyo, Japan). The samples (1 mg/ml) were prepared in 50% acetonitrile containing 0.1% formic acid solvent and a drop of the diluted protein suspension solutions (15 μl) was poured on a polycarbon film supported on a copper grid and dried at ambient temperature. The samples were examined on TEM at an accelerating voltage of 200 kV. The sizes of the protein nano-structures were obtained using iTEM software (Olympus, Münster, Germany). The radius of the diffraction rings in SAED pattern is the inverse of the d-spacing of the associated lattice planes and radii of the rings were determined using iTEM software.

### Statistical analysis

The results were expressed as a mean ± SD and compared statistically at p ≤ 0.05, using one-way analysis of variance (ANOVA) with Tukey’s post hoc test (Version 17, Minitab Inc., State College, USA).

## Result and discussion

**Figure 1:**
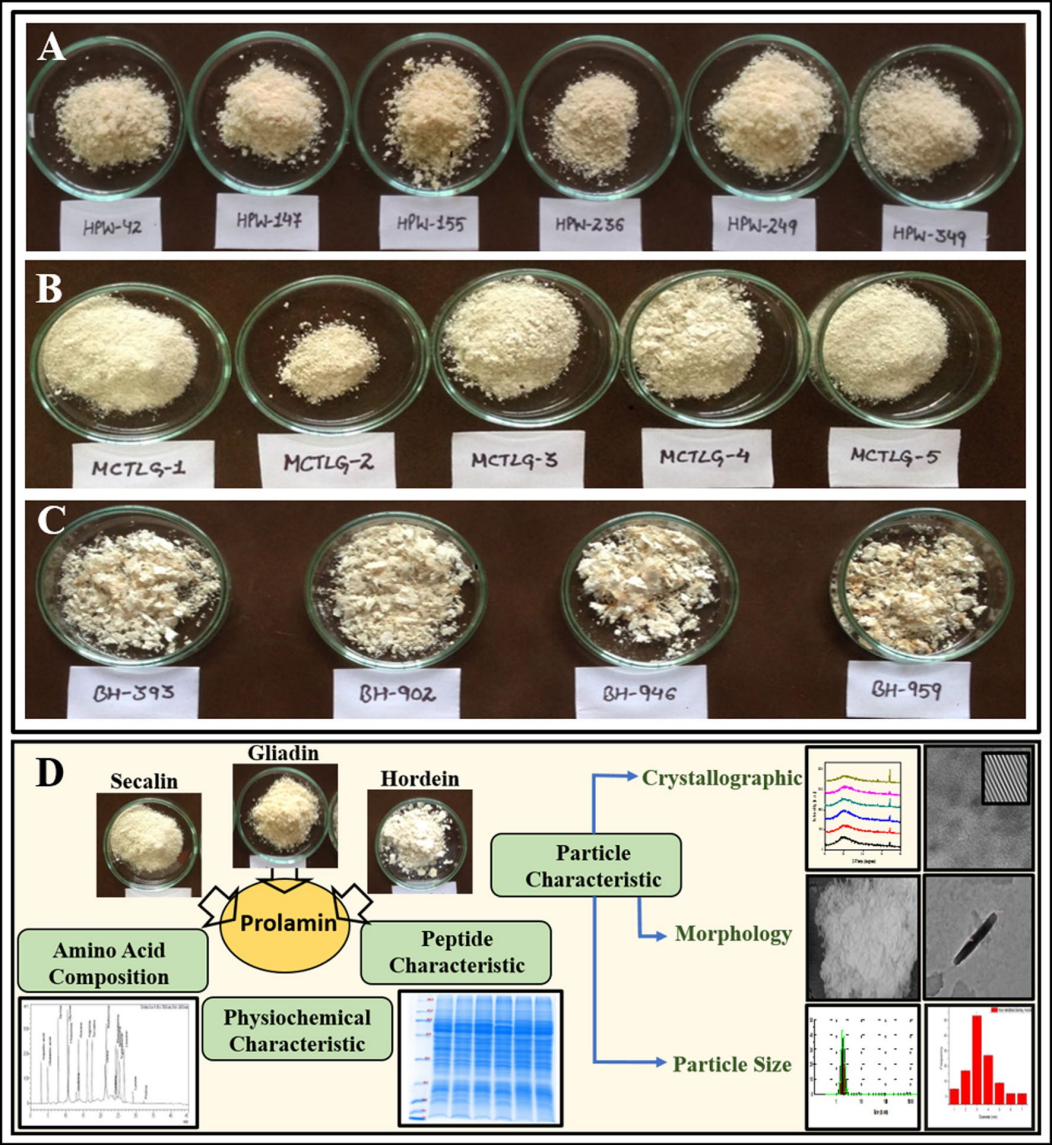
Images of prolamin freeze-dried powder (**A**) Gliadin, (**B**) Secalin and (**C**) Hordein extracted with DuPont et al., (2005) extraction protocol. While (**D**) represents graphical representation of the investigations conducted on prolamin fraction from wheat, rye and barley.

Wheat, rye and barley belonging to the Triticeae tribe, rich in gluten were extracted using DuPont et al.^11^ protocol to obtain prolamin fractions known as gliadins in wheat, secalins in rye and hordeins in barley. We showed the properties of extracted freeze-dried prolamins fraction using different analytical techniques. The extracted prolamin fractions contained prolamins as a major component but with cross contamination of Alb + Glo which needs to be considered while interpreting the results. The color of freeze-dried powder of gliadins, hordeins and secalins fraction was light cream (Fig. 1).

### Physiochemical characteristics

The protein content (PC) of the freeze-dried prolamin fractions extracted from wheat, rye and barley followed a decreasing order as gliadins (64.94–70.26%), secalins (47.50–60.70%) and hordeins (34.06–45.17%) respectively (Table S1). Among the cultivars, the gliadins fraction from HPW-349 contained the highest PC while hordein fraction from BH-959 contained the lowest. The PC of prolamin fractions was found to vary significantly (p ≤ 0.05). DuPont et al.^11^ reported 40.1% protein content in extracted gliadins fraction which was slightly lowered than our findings. He et al.^12^ reported higher protein content of dialyzed gliadin (85%) using alcohol extraction protocol and conversion factor of 6.25 instead of 5.7 used in this study. However, Schalk et al.^13^ also reported the protein content of dialysed gliadin, secalin and hordein to be 93.5%, 89.4% and 87.3% respectively using 5.7 conversion factor and alcohol extraction protocol. The difference in the crude protein content may be due to difference in extraction protocol, dialysis performed and also to some extent genetic makeup of cultivars.

The color characteristics of the prolamin fractions were explained via CIE color values (L*, a*, b*, Hue angle (H^o^) and Chroma (C*). L* value which indicates the lightness was found to be in the range 53.13–68.12 (Table S1). In the investigated cereals, the L* value for hordein fraction from BH-902 was found to be significantly (p ≤ 0.05) higher while lowest for secalin fraction from MCTLG-1. The dark color (lower L* value) of the secalins fraction might be due to the presence of pigment and oxidative changes as compared to hordeins and gliadins fraction^44^. In case of coordinates a* and b*, the observed positive values for prolamin fraction fall in the range 0.53–2.06 and 6.90–13.93 implying that prolamin fractions had red and yellow tinge respectively. For the investigated cultivars, the a* value was found to be the highest for gliadins fraction (1.53–2.06) and the lowest for hordeins fraction (0.53–1.28). On the other hand, the higher b* values for gliadins fraction (9.68–13.93) reflected more yellow tint in comparison to secalins fraction (6.90–10.13) and hordeins fraction (7.41–9.61). On the whole, a significant (p ≤ 0.05) difference in a* and b* values were observed among the prolamin fractions at inter as well as at intra-cultivars levels.

### Polypeptide characteristics

The gliadins, secalins and hordeins fraction were analysed for their electrophoretic pattern on SDS-PAGE (Fig. S1). The total number of polypeptides ranged from 16–21 in wheat cultivars, 14–16 in rye cultivars and 16–20 in barley cultivars. The prolamins polypeptides were sub-categorized as ω-gliadin, α/β–gliadin and γ-gliadin in wheat cultivars; C-hordein and γ/B-hordein in barley cultivars and γ-75 k-secalin, ω-secalin and γ-40 k-secalin in rye cultivars following Schalk et al.^13^. The High Molecular Weight (HMW) proteins in wheat, rye, and barley comprised of HMW- Non Gliadin components (HMW-NG), HMW-secalin, D-Hordein respectively. Medium Molecular Weight (MMW) proteins were ω-gliadin (wheat), ω-secalin (rye), and C-hordein (barley), while Low Molecular Weight (LMW) proteins were monomeric proteins (α/β–gliadin, γ-gliadin in wheat, γ-40 k-secalin in rye and γ-hordein in barley) and polymeric proteins (LMW-GS in wheat, γ-75 k-secalin in rye and B-hordein in barley).

Minor traces of HMW-NG, HMW-secalin and D-hordein were observed in wheat, rye and barley cultivars respectively. These high molecular weight bands consisted of 2–4 polypeptides of M_w_ 91.60–122.77 kDa in wheat cultivars and a single polypeptide of M_w_ 93.37–106.37 kDa in rye and single polypeptide of M_w_ 84.55–97.26 kDa in barley cultivars (Figure S1 a-c). Densitometric analysis (Table 1 and Figure S4) showed that the relative proportion of these HMW proteins varied from 2.08–4.82% in gliadins, 2.43–3.72% in secalins and 2.17–5.17% in hordeins. Schalk et al.^13^ also reported SDS-PAGE of gliadin where HMW-NG accounted for 2.8%.

**Table 1:** The relative proportion of gliadin, secalin and hordein protein in SDS-PAGE and their classification according to molecular weight under reducing conditions.

Prolamins	HMW	MMW (S-poor)	LMW (S-rich)	Albumin + Globulin
***Gliadin***				
HPW-42	2.83 ± 0.50^BC^	9.78 ± 0.81^E^	74.27 ± 2.36^A^	13.13 ± 2.67^DE^
HPW-147	2.08 ± 0.66^C^	15.06 ± 0.77^BCDE^	67.42 ± 1.85^ABC^	15.44 ± 0.42^BCDE^
HPW-155	3.16 ± 0.97^ABC^	22.54 ± 0.46^AB^	60.98 ± 1.22^BCDE^	13.31 ± 1.72^DE^
HPW-236	3.18 ± 0.73^ABC^	21.72 ± 1.96^ABC^	60.82 ± 0.74^BCDE^	14.33 ± 1.88^CDE^
HPW-249	4.82 ± 0.03^AB^	12.94 ± 1.09^DE^	72.26 ± 3.79^A^	9.98 ± 2.67^E^
HPW-349	3.37 ± 0.66^ABC^	14.48 ± 0.94^CDE^	70.54 ± 1.23^AB^	11.62 ± 2.82^DE^
***Secalin***				
MCTLG-1	2.67 ± 0.42^BC^	16.50 ± 1.41^DEF^	(17.44 ± 2.52** + 41.82 ± 2.16*) 59.26 ± 0.36^CDE^	21.57 ± 2.19^AB^
MCTLG-2	3.44 ± 0.81^ABC^	13.08 ± 2.97^F^	(11.65 ± 1.49** + 48.37 ± 2.28) 60.01 ± 3.78^CDE^	23.47 ± 0.01^A^
MCTLG-3	3.72 ± 0.09^ABC^	16.07 ± 4.23^DEF^	(18.77 ± 1.11** + 38.67 ± 3.63*) 57.44 ± 4.73^CDEF^	22.77 ± 0.40^A^
MCTLG-4	2.46 ± 0.20^C^	15.52 ± 0.83^DEF^	(14.20 ± 2.48** + 46.90 ± 2.82*) 61.10 ± 0.34^BCDE^	20.98 ± 1.30^ABC^
MCTLG-5	2.43 ± 1.06^C^	13.30 ± 1.81^F^	(18.22 ± 1.99** + 48.22 ± 2.21*) 66.45 ± 0.23^ABCD^	17.82 ± 0.98^ABCD^
***Hordein***				
BH-393	2.17 ± 0.11^C^	13.46 ± 0.07^DE^	61.96 ± 2.22^BCDE^	22.40 ± 2.25^A^
BH-902	4.72 ± 0.17^AB^	23.89 ± 0.43^A^	48.96 ± 1.29^F^	22.43 ± 1.03^A^
BH-946	3.77 ± 0.08^ABC^	20.15 ± 3.57^ABCD^	54.22 ± 3.83^EF^	21.86 ± 0.34^AB^
BH-959	5.17 ± 0.23^A^	16.56 ± 1.98^ABCDE^	57.15 ± 3.49^DEF^	21.11 ± 1.28^ABC^

** refers to γ-75 k-secalin fraction and * refers to γ-40 k-secalin.

Mean ± SD with different superscripts in column differ significantly (*p* ≤ 0.05); *n* = 3 for each treatment.

The MMW proteins comprised of sulphur poor monomeric protein namely ω-gliadin which distinguished into 3–6 polypeptides of M_w_ 52.91–88.82 kDa in gliadin, 2–3 polypeptides (M_W_ 55.60–84.64 kDa) in C-hordein and a single polypeptide (M_W_ 45.09–49.75 kDa) in ω-secalin. The relative proportion of MMW protein (Table 1), ranged from 9.78–23.89% of total extracted prolamins with C-hordein (13.46–23.89%); ω-secalin (13.08–16.50%) and ω-gliadin (9.78–22.54%). This fraction holds repetitive domain of prolamin and are usually rich in β-turn^15,45,46^. The highest relative proportion of MMW proteins was observed in hordeins from BH-902 (23.89%) and lowest in gliadins from HPW-42 (9.78%). A significant (p ≤ 0.05) difference in the values of MMW proteins was observed.

According to Lexhaller et al.^47^, the LMW protein covers the monomeric peptides (identified as α/β-gliadins, γ-gliadins in wheat; γ-40 k-secalins in rye and γ-hordeins in barley) and polymeric peptide (recognized as LMW-GS in wheat, γ-75 k-secalins in rye and B-hordeins in barley). The LMW protein in wheat cultivars comprised of α/β-gliadins, γ-gliadins together with LMW-GS, distributed in MW range between 25.97–50.06 kDa, while γ/B-hordein dispersed in the MW range of 25.01–54.75 kDa in barley. The secalin consisted of γ-40 k-secalins and γ-75 k-secalins with MW range between 25.14–41.11 kDa and 52.85–67.70 kDa respectively. The relative proportion of LMW protein ranged from 48.96–74.27% of extracted prolamin with higher amount in gliadins (60.82–74.27%), followed by secalins (57.44–66.45%) and hordeins (48.96–61.96%) as elaborated in Table 1. The highest relative proportion of LMW proteins was observed in gliadins from HPW-42 (74.27%) and the lowest in hordeins from BH-902 (48.96%). The LMW protein of secalins fraction comprises of γ-75 k-secalins and γ-40 k-secalins with relative proportion of 11.65–18.77% and 38.67–48.32% respectively. The γ-75 k-secalins were obtained in high concentration in rye cultivar MCTLG-3 and in low concentration in rye cultivar MCTLG-2 whereas the highest concentration of γ-40 k-secalins was observed in rye cultivar MCTLG-2 and the lowest in MCTLG-3.

The earlier studies have reported molecular weights in the vicinity of 80–120 kDa for HMW-GS, 43–68 kDa for ω- gliadin and 32–45 kDa for α/β-, γ-gliadins^13,37^; about 100 kDa for D-hordein, 55–86 kDa as C-hordein and 30–50 kDa B-hordein^13,16,29^ and 95–105 kDa for HMW-secalins, 33–37 kDa for 40 k-γ-secalins and 54–66.2 kDa for 75 k-γ-secalins^13,26,37^. The slight variation in MW in the present study might be due to the genetic makeup of the cultivars, agro-environmental conditions and protocol employed for extraction of prolamin. Wieser and Kieffer^22^ reported ω-gliadin as 9.85–18.38% and α/β-, γ- gliadin as 70.94–95.9% of total extracted gliadin. Siddiqi et al.^32^ reported the proportion of HMW-GS in the range of 0.83–2.99%, and α/β-, γ- gliadin varying between 72.16–84.67% in fourteen Indian wheat cultivars which is very close to our present finding, but lower proportion of ω-gliadin (1.53–5.31%). The relative proportion of the HMW group ranged from 2–4% in hordein, 7% in secalin and 10% in gliadin; sulphur poor ω-prolamin about 10–20% in hordein, while 11% in secalin and gliadin; whereas the LMW prolamin fraction constituted about 70–80% of total prolamins in gliadin, secalin and hordein^16^. Differences in the proportion of various prolamin fractions may be due to genetic make-up of cultivars, different extraction procedure followed and so on. The extraction of prolamins from different plant sources is influenced by the extraction solvent used-especially the alcohol percentage, particle size of sample, sample to solvent ratio, number of extractions performed and their timings, extraction temperature, pH, use of reducing agents etc.^32,37,48^.

The prolamin (DuPont extraction protocol^11^) fractions were found to be contaminated with metabolically active proteins (Alb + Glo) having molecular weight range between 9.56–24.53 kDa, 17.78–23.93 kDa and 14.96–22.84 kDa for gliadins, secalins and hordeins respectively. Most of the albumin and globulin have a molecular weight well below 25 kDa^11,32,49^. Densitometric evaluation (Table 1) revealed the relative proportion of Alb + Glo protein varied between 9.98–21.57%. Highest proportion of Alb + Glo was found in hordeins (21.11–22.43%), followed by secalins fraction (17.82–21.57%) and gliadins (9.98–16.97%) fractions.

Genetic proximity of various accessions in the present study was further evaluated through Jaccard Similarity Matrix by means of Un-weighted Pair Group Method with Arithmetic Averages (UPGMA) to construct a dendrogram (Figure S4). Three distinct groups could be identified from the dendrogram of gliadins, secalins and hordeins fraction; moreover, gliadins and hordeins fractions were closer to each other than secalins fraction. In case of gliadins fraction, group-I had two accessions where HPW-42 was found to be dissimilar from rest of the other extracted gliadins with similarity coefficient of 0.58; Group-II had two accessions of closely resembled cluster of HPW-147, HPW-155, HPW-236 and HPW-249, HPW-349 with similarity coefficient of 0.76; while Group-III had further two accessions of closely resembled cluster of HPW-147, HPW-155 with similarity coefficient of 0.85 and other HPW-236 with 0.75 similarity coefficient. On the other hand, hordeins fraction had three accessions where BH-902 and BH-393 was observed to be closely resembled with similarity coefficient of 0.82 while, BH-959 and BH-946 are dissimilar from rest of the other with similarity coefficient of 0.70 and 0.74 respectively. Unlike closely resembled gliadins and hordeins fraction, the secalins fraction was distinguished into two accessions with closely resembled clusters of MCTLG-4, MCTLG-5 with similarity coefficient of 0.78 while another cluster of MCTLG-2, MCTLG-3 and MCTLG-1 where MCTLG-2 and MCTLG-3 shared similarity index of 0.88.

### Amino Acid Composition

**Figure 2:**
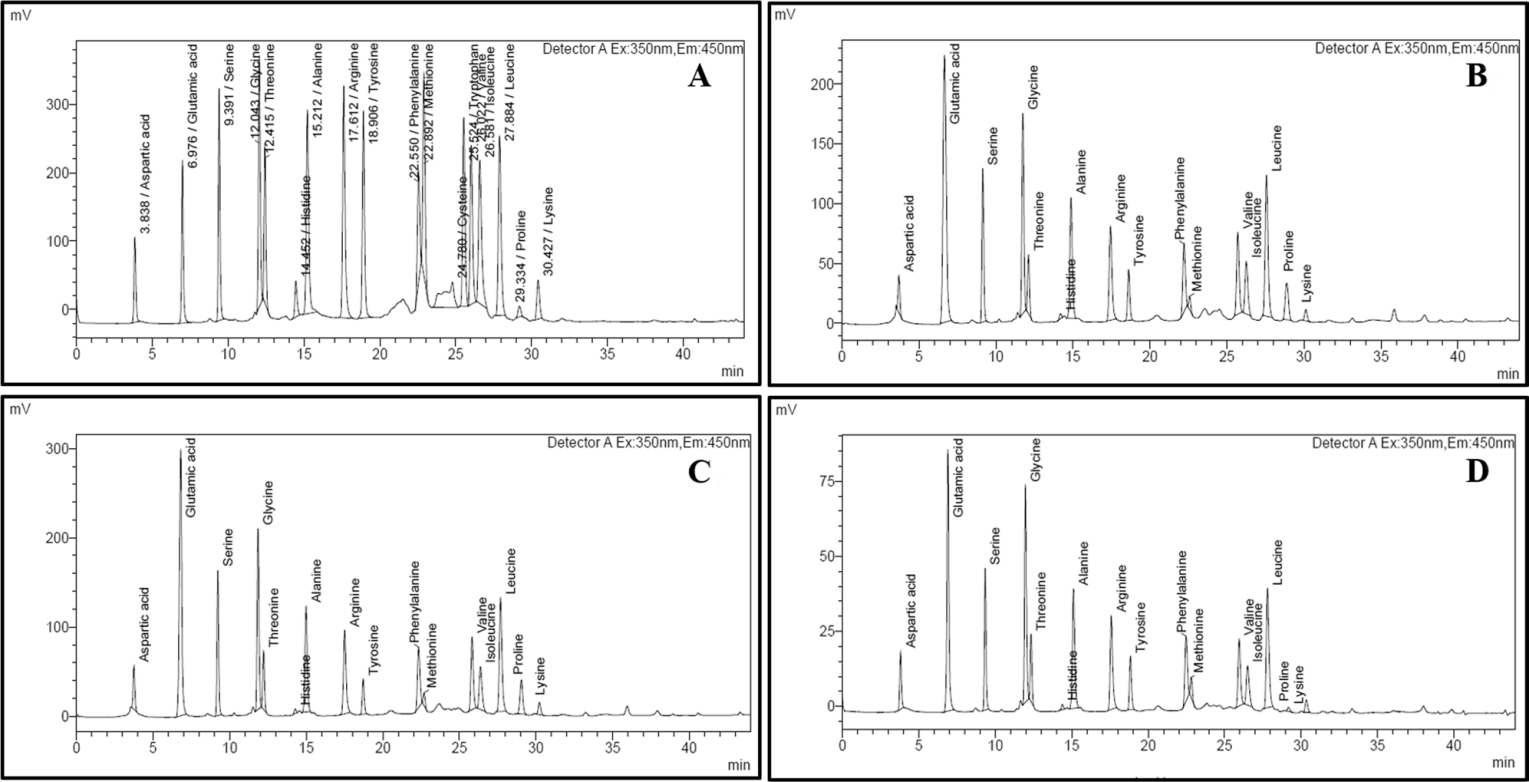
HPLC Chromatograms displaying (**A**) 500 mmol standard mixture of 18 Amino acids; (**B**) Gliadin, HPW-42 (**C**) Secalin, MCTLG-5, (**D**) Hordein, BH-393.

The amino acid composition of prolamin fraction from wheat, rye and barley cultivars is presented in Tables 2, 3, 4 and Fig. 2 & S3. The conventional methodology for determining amino acid composition, which included acid digestion followed by chromatographic analysis, was examined. Some amino acids undergo a various kind of chemical changes during acid hydrolysis. Tryptophan is destroyed; serine, cysteine, and threonine are partially destroyed; methionine is oxidized; tyrosine is halogenated or oxidized; valine and isoleucine require 72 h for complete hydrolysis and are only hydrolysed to about 50–70% at 110 °C in 24 h^40^. As no correction factors for these phenomena were determined in the present manuscript, the amino acid levels reported on are estimated values, rather than representing an exact quantitation.

**Table 2:** Essential Amino Acid (EAA) Composition (g amino acid/100 g protein) of Gliadin, Secalin and Hordein Fractions.

Prolamin/Cultivar	His	Thr	Phe	Met	Val	Ileu	Leu	Lys	TEAA
***Gliadin***									
HPW-42	1.52 ± 0.46^ABC^	2.30 ± 0.29^AB^	5.69 ± 0.44^AB^	0.71 ± 0.00^A^	5.12 ± 0.29^AB^	3.48 ± 0.34^A^	7.05 ± 0.33^A^	0.70 ± 0.40^A^	27.34 ± 1.95^ABC^
HPW-147	2.14 ± 0.08^AB^	2.88 ± 0.29^AB^	6.48 ± 0.14^AB^	0.91 ± 0.22^A^	5.08 ± 0.65^AB^	3.63 ± 0.55^A^	7.01 ± 0.55^A^	0.52 ± 0.15^A^	28.59 ± 1.49^ABC^
HPW-155	1.27 ± 0.09^BC^	2.66 ± 0.61^AB^	5.94 ± 0.79^AB^	1.04 ± 0.03^A^	5.28 ± 0.51^AB^	3.93 ± 0.98^A^	6.50 ± 0.71^A^	0.77 ± 0.07^A^	27.08 ± 2.46^ABC^
HPW-236	0.83 ± 0.53^C^	2.80 ± 0.41^AB^	5.68 ± 0.37^AB^	0.75 ± 0.29^A^	4.67 ± 0.60^B^	3.54 ± 0.43^A^	6.82 ± 0.07^A^	0.79 ± 0.04^A^	26.09 ± 0.58^C^
HPW-249	2.17 ± 0.24^AB^	2.62 ± 0.61^AB^	5.82 ± 0.04^AB^	0.87 ± 0.27^A^	6.08 ± 0.60^AB^	4.53 ± 0.15^A^	7.20 ± 0.40^A^	0.72 ± 0.24^A^	30.10 ± 1.07^ABC^
HPW-349	1.41 ± 0.57^ABC^	1.95 ± 0.08^B^	5.19 ± 0.19^B^	0.69 ± 0.06^A^	5.42 ± 0.52^AB^	4.18 ± 0.04^A^	6.70 ± 0.20^A^	0.69 ± 0.43^A^	26.47 ± 2.54^BC^
***Secalin***									
MCTLG-1	1.68 ± 0.14^ABC^	3.31 ± 0.58^AB^	6.63 ± 0.32^AB^	0.69 ± 0.22^A^	5.28 ± 0.25^AB^	3.40 ± 0.46^A^	6.60 ± 0.23^A^	0.75 ± 0.02^A^	28.61 ± 0.30^ABC^
MCTLG-2	1.85 ± 0.10^ABC^	2.60 ± 0.12^AB^	5.95 ± 0.14^AB^	0.75 ± 0.31^A^	5.24 ± 0.45^AB^	3.20 ± 0.17^A^	6.26 ± 0.06^A^	0.47 ± 0.05^A^	26.66 ± 1.53^BC^
MCTLG-3	2.13 ± 0.29^ABC^	3.12 ± 0.57^AB^	6.28 ± 0.30^AB^	1.30 ± 0.33^A^	5.66 ± 0.54^AB^	3.38 ± 0.43^A^	6.43 ± 0.02^A^	0.93 ± 0.19^A^	28.80 ± 1.04^ABC^
MCTLG-4	2.64 ± 0.40^A^	3.42 ± 0.14^AB^	7.09 ± 0.85^AB^	1.51 ± 0.03^A^	6.87 ± 0.84^A^	3.57 ± 0.69^A^	6.24 ± 0.82^A^	0.58 ± 0.25^A^	31.48 ± 2.00^ABC^
MCTLG-5	1.67 ± 0.13^ABC^	2.77 ± 0.26^AB^	5.67 ± 0.96^AB^	0.78 ± 0.40^A^	5.99 ± 0.69^AB^	3.67 ± 0.29^A^	6.77 ± 0.17^A^	0.66 ± 0.17^A^	27.82 ± 2.53^ABC^
***Hordein***									
BH-393	2.69 ± 0.47^A^	3.87 ± 0.52^A^	6.93 ± 0.63^AB^	1.64 ± 0.26^A^	6.01 ± 0.39^AB^	3.82 ± 0.22^A^	6.86 ± 0.69^A^	0.96 ± 0.60^A^	32.86 ± 1.25^AB^
BH-902	1.49 ± 0.03^ABC^	3.82 ± 0.59^A^	6.86 ± 0.53^AB^	1.26 ± 0.78^A^	4.38 ± 0.14^B^	3.58 ± 0.57^A^	6.56 ± 0.26^A^	0.93 ± 0.32^A^	29.10 ± 0.69^ABC^
BH-946	2.24 ± 0.31^AB^	3.41 ± 0.24^AB^	5.91 ± 0.82^AB^	1.68 ± 0.19^A^	4.81 ± 0.75^AB^	3.86 ± 0.17^A^	7.03 ± 0.92^A^	1.12 ± 0.56^A^	30.52 ± 1.91^ABC^
BH-959	2.27 ± 0.35^AB^	3.01 ± 0.66^AB^	7.79 ± 1.18^A^	1.11 ± 0.59^A^	5.63 ± 0.36^AB^	4.34 ± 0.63^A^	7.14 ± 0.42^A^	1.64 ± 0.39^A^	33.55 ± 1.00^A^

His– Histidine, Thr – Threonine, Val – Valine, Met – Methionine, Phe – Phenylalanine, Ileu – Isoleucine, Leu – Leucine, Lys – Lysine and TEAA – Total Essential Amino Acid.

Mean ± SD with different superscripts in column differ significantly (*p* ≤ 0.05); *n* = 3 for each treatment.

**Table 3:** Non-Essential Amino Acid (NEAA) Composition (g amino acid/100 g protein) of Gliadin, Secalin and Hordein Fractions.

Prolamin/Cultivar	Asn + Asp	Gln + Glu	Ser	Gly	Ala	Arg	Tyr	Pro	NEAA
***Gliadin***									
HPW-42	2.69 ± 0.35^C^	38.33 ± 1.46^A^	6.12 ± 0.36^A^	3.07 ± 0.35^A^	2.30 ± 0.14^A^	3.74 ± 0.21^AB^	3.52 ± 0.42^AB^	13.66 ± 1.13^ABC^	72.65 ± 1.95^ABC^
HPW-147	4.41 ± 0.13^ABC^	39.34 ± 1.79^A^	6.45 ± 0.57^A^	2.84 ± 0.06^A^	2.31 ± 0.16^A^	3.08 ± 0.56^AB^	3.60 ± 0.83^AB^	9.34 ± 0.51^BC^	71.41 ± 1.49^ABC^
HPW-155	4.95 ± 0.80^AB^	41.90 ± 2.57^A^	6.26 ± 0.26^A^	2.34 ± 0.54^A^	2.25 ± 0.22^A^	2.87 ± 0.26^AB^	3.35 ± 0.75^AB^	8.67 ± 1.13^BC^	72.92 ± 2.46^ABC^
HPW-236	4.42 ± 0.16^ABC^	39.14 ± 1.48^A^	5.72 ± 0.48^A^	3.00 ± 0.03^A^	2.77 ± 0.61^A^	2.98 ± 0.23^AB^	3.38 ± 0.37^AB^	12.70 ± 1.00^ABC^	73.91 ± 0.58^A^
HPW-249	3.97 ± 0.63^ABC^	39.48 ± 0.79^A^	5.26 ± 0.65^A^	2.71 ± 0.47^A^	2.53 ± 0.58^A^	3.08 ± 0.05^AB^	3.44 ± 0.73^AB^	9.53 ± 0.67^ABC^	69.90 ± 1.07^ABC^
HPW-349	4.27 ± 0.29^ABC^	39.42 ± 1.79^A^	6.28 ± 0.03^A^	2.19 ± 0.72^A^	2.45 ± 0.18^A^	3.53 ± 0.32^AB^	3.83 ± 0.45^AB^	11.79 ± 1.09^ABC^	73.53 ± 2.54^AB^
***Secalin***									
MCTLG-1	3.24 ± 0.31^BC^	35.14 ± 1.52^AB^	6.69 ± 0.10^A^	3.59 ± 0.22^A^	2.62 ± 0.39^A^	3.69 ± 0.18^AB^	3.08 ± 0.15^AB^	13.62 ± 0.94^ABC^	71.39 ± 0.30^ABC^
MCTLG-2	3.35 ± 0.32^BC^	38.00 ± 2.18^A^	6.34 ± 0.19^A^	2.94 ± 0.56^A^	2.52 ± 0.26^A^	3.41 ± 0.29^AB^	2.91 ± 0.10^B^	14.22 ± 0.73^AB^	73.34 ± 1.53^AB^
MCTLG-3	2.99 ± 0.40^C^	38.04 ± 1.51^AB^	6.46 ± 0.37^A^	2.69 ± 0.20^A^	2.29 ± 0.30^A^	3.08 ± 0.16^AB^	2.81 ± 0.24^B^	12.41 ± 1.23^ABC^	71.20 ± 1.03^ABC^
MCTLG-4	3.50 ± 0.42^BC^	35.57 ± 1.38^AB^	7.05 ± 1.21^A^	2.99 ± 0.62^A^	2.64 ± 0.19^A^	3.96 ± 0.34^AB^	3.27 ± 0.40^AB^	9.11 ± 2.37^BC^	68.52 ± 2.00^ABC^
MCTLG-5	3.20 ± 0.77^BC^	39.37 ± 2.07^A^	5.880 ± 0.90^A^	2.88 ± 0.02^A^	2.00 ± 0.28^A^	2.56 ± 0.38^B^	3.02 ± 0.19^B^	13.49 ± 0.81^ABC^	72.18 ± 2.53^ABC^
***Hordein***									
BH-393	4.81 ± 0.27^AB^	29.46 ± 1.47^BC^	6.81 ± 0.27^A^	3.95 ± 0.67^A^	3.20 ± 0.25^A^	3.96 ± 0.29^AB^	4.93 ± 0.07^A^	10.09 ± 2.72^ABC^	67.14 ± 1.25^BC^
BH-902	5.56 ± 0.09^A^	29.02 ± 2.18^BC^	6.01 ± 0.02^A^	4.02 ± 0.76^A^	3.21 ± 0.24^A^	4.13 ± 0.54^AB^	4.19 ± 0.30^AB^	14.97 ± 1.60^A^	70.90 ± 0.69^ABC^
BH-946	5.46 ± 0.22^A^	30.93 ± 1.46^BC^	6.47 ± 0.22^A^	3.86 ± 0.54^A^	3.72 ± 093^A^	4.36 ± 0.86^A^	4.48 ± 0.70^AB^	10.66 ± 0.88^ABC^	69.48 ± 1.91^ABC^
BH-959	4.35 ± 0.61^ABC^	28.25 ± 1.27^C^	6.11 ± 0.13^A^	3.65 ± 0.19^A^	3.24 ± 0.89^A^	3.46 ± 0.65^AB^	3.85 ± 0.44^AB^	14.18 ± 1.61^AB^	66.45 ± 1.00^C^

Asn + Asp – Asparagine + Aspartic Acid, Gln + Glu – Glutamine + Glutamic Acid, Ser – Serine, Gly – Glycine, Arg – Arginine, Ala – Alanine, Tyr – Tyrosine, Pro – Proline, TNEAA – Total Non- Essential Amino Acid.

Mean ± SD with different superscripts in column differ significantly (*p* ≤ 0.05); *n* = 3 for each treatment.

**Table 4:** IMGT (ImMunoGeneTics) Amino Acid Classification (Pommie et al.,2004) of gliadin, secalin and hordein fractions (g amino acid/100 g protein) from different cultivars.

Prolamin/Cultivar	Hydrophobic Amino Acid				Hydrophillic Amino Acid			Neutral Amino Acid			
	Aliphatic	S-containing	Aromatic	Total Hydrophobic	Basic	Acidic	Total Hydrophillic	Non-Polar	Polar (Hydroxyl AA)	Aromatic	Total Neutral
***Gliadin***											
HPW-42	19.39 ± 0.09^A^	0.71 ± 0.00^A^	5.69 ± 0.44^AB^	25.79 ± 0.53^A^	6.07 ± 0.89^ABC^	41.02 ± 1.82^ABCD^	47.08 ± 0.93^ABCDE^	15.96 ± 0.84^ABC^	7.65 ± 0.82^BCD^	3.52 ± 0.42^AB^	27.12 ± 0.40^ABCD^
HPW-147	18.80 ± 2.30^A^	0.91 ± 0.22^A^	6.48 ± 0.14^AB^	26.18 ± 2.22^A^	5.67 ± 0.07^BC^	43.74 ± 1.66^AB^	49.41 ± 1.60^ABC^	12.22 ± 0.80^BC^	8.59 ± 0.65^ABC^	3.60 ± 0.83^AB^	24.41 ± 0.63^BCD^
HPW-155	18.59 ± 2.46^A^	1.04 ± 0.03^A^	5.94 ± 0.79^AB^	25.57 ± 3.23^A^	5.36 ± 0.82^C^	46.86 ± 1.77^A^	52.22 ± 2.59^A^	11.33 ± 1.74^C^	7.53 ± 0.35^CD^	3.35 ± 0.75^AB^	22.21 ± 0.64^D^
HPW-236	18.02 ± 1.33^A^	0.75 ± 0.29^A^	5.68 ± 0.37^AB^	24.46 ± 1.41^A^	6.56 ± 0.68^ABC^	43.55 ± 1.64^ABC^	50.11 ± 2.32^AB^	15.51 ± 0.59^ABC^	6.55 ± 0.04^D^	3.38 ± 0.37^AB^	25.44 ± 0.91^ABCD^
HPW-249	20.88 ± 0.30^A^	0.87 ± 0.27^A^	5.82 ± 0.04^AB^	27.58 ± 0.54^A^	5.95 ± 0.82^ABC^	43.45 ± 0.16^ABC^	49.40 ± 0.66^ABC^	12.15 ± 0.06^BC^	7.43 ± 0.41^CD^	3.44 ± 0.73^AB^	23.02 ± 1.20^CD^
HPW-349	19.83 ± 1.00^A^	0.69 ± 0.06^A^	5.19 ± 0.19^B^	25.71 ± 1.13^A^	5.33 ± 0.98^C^	43.69 ± 2.08^ABC^	49.02 ± 1.10^ABCD^	13.74 ± 1.02^ABC^	7.70 ± 0.54^BCD^	3.83 ± 0.45^AB^	25.27 ± 0.03^BCD^
***Secalin***											
MCTLG-1	18.97 ± 0.31^A^	0.69 ± 0.22^A^	6.63 ± 0.32^AB^	26.28 ± 0.22^A^	6.96 ± 0.63^ABC^	38.38 ± 1.84^BCDE^	45.34 ± 1.21^BCDE^	16.92 ± 1.52^ABC^	8.37 ± 0.24^ABCD^	3.08 ± 0.15^AB^	28.38 ± 1.43^AB^
MCTLG-2	18.11 ± 0.96^A^	0.75 ± 0.31^A^	5.95 ± 0.14^AB^	24.81 ± 1.41^A^	5.93 ± 0.25^ABC^	41.34 ± 2.50^ABCD^	47.27 ± 2.25^ABCDE^	16.83 ± 0.85^ABC^	8.18 ± 0.09^ABCD^	2.91 ± 0.10^B^	27.92 ± 0.84^ABC^
MCTLG-3	18.56 ± 0.82^A^	1.30 ± 0.33^A^	6.28 ± 0.30^AB^	26.13 ± 0.19^A^	5.91 ± 0.09^ABC^	41.03 ± 1.10^ABCD^	46.94 ± 1.19^ABCDE^	15.53 ± 1.79^ABC^	8.59 ± 0.66^ABC^	2.81 ± 0.24^B^	26.92 ± 1.38^ABCD^
MCTLG-4	20.64 ± 0.37^A^	1.51 ± 0.03^A^	7.09 ± 0.85^AB^	29.24 ± 1.19^A^	6.20 ± 1.07^ABC^	39.07 ± 0.96^BCDE^	45.28 ± 0.11^BCD^	12.53 ± 2.50^BC^	9.69 ± 0.81^A^	3.27 ± 0.40A^B^	25.48 ± 1.30^ABCD^
MCTLG-5	18.86 ± 1.54^A^	0.79 ± 0.40^A^	5.68 ± 0.96^AB^	25.33 ± 2.90^A^	5.40 ± 0.43^ABC^	42.57 ± 2.84^ABC^	47.97 ± 2.41^ABCD^	16.21 ± 1.07^ABC^	7.46 ± 0.77^CD^	3.02 ± 0.19^B^	26.70 ± 0.49^ABCD^
***Hordein***											
BH-393	20.65 ± 0.37^A^	1.64 ± 0.26^A^	6.93 ± 0.63^AB^	29.22 ± 0.74^A^	8.11 ± 1.02^ABC^	34.27 ± 1.75^DE^	42.38 ± 2.76^DE^	13.96 ± 3.23^ABC^	9.50 ± 0.20^AB^	4.93 ± 0.07^A^	28.39 ± 3.50^AB^
BH-902	18.65 ± 0.37^A^	1.26 ± 0.78^A^	6.86 ± 0.53^AB^	26.78 ± 0.11^A^	8.16 ± 0.84^ABC^	34.58 ± 2.27^DE^	42.74 ± 1.43^CDE^	18.79 ± 1.01^A^	7.50 ± 0.01^CD^	4.19 ± 0.30^AB^	30.48 ± 1.32^A^
BH-946	20.06 ± 2.36^A^	1.68 ± 0.19^A^	5.91 ± 0.82^AB^	27.65 ± 1.35^A^	8.70 ± 0.18^A^	36.39 ± 1.68^CDE^	45.09 ± 1.51^BCDE^	14.07 ± 0.64^ABC^	8.71 ± 0.10^ABC^	4.48 ± 0.70^AB^	27.25 ± 0.16^ABCD^
BH-959	20.57 ± 0.09^A^	1.11 ± 0.59^A^	7.79 ± 1.18^A^	29.46 ± 0.50^A^	8.52 ± 1.09^AB^	32.60 ± 1.88^E^	41.12 ± 0.80^E^	17.19 ± 0.95^AB^	8.38 ± 0.21^ABCD^	3.85 ± 0.44^AB^	29.42 ± 0.30^AB^

Aliphatic amino acid: Alanine + Valine + Isoleucine + Leucine, S-containing amino acids: Cysteine + Methionine, Basic amino acids: Arginine + Lysine + Histidine, Acidic amino acids: Aspartic + Glutamic acid, Hydrophobic Aromatic amino acids: Phenylalanine + Tryptophan, Non-Polar amino acids: Glycine + Proline, Polar Hydroxy amino acids: Serine + Threonine, Neutral Aromatic amino acids: Tyrosine.

Total Hydrophobic amino acid: Alanine + Cysteine + Valine + Methionine + Tryptophan + Phenylalanine + Isoleucine + Leucine; Total Hydrophillic amino acid: Aspartic Acid + Glutamic Acid + Arginine + Lysine; Neutral amino acid: Serine + Histidine + Glycine + Threonine + Tyrosine + Proline.

Mean ± SD with different superscripts in column differ significantly (*p* ≤ 0.05); *n* = 3 for each treatment.

Among EAA, the foremost amino acids were found to be Phe, Leu and Val which constituted about 5.19–7.79%, 6.24–7.20% and 4.38–6.87% respectively for different prolamins. Other EAA were His (0.83–2.69%), Thr (1.95–3.87%), Lys (0.47–1.64%), Ileu (3.20–4.53%), and Met (0.69–1.68%). His, Thr, Val and Phe contents were observed to vary significantly (p ≤ 0.05) except among Thr and Phe content of secalin fraction where non-significant (p ≥ 0.05) difference was observed. While, Ileu, Met, Leu and Lys varied non-significantly (p ≥ 0.05) among different cereals and within their respective cultivars.

The estimated amount of His and Phe was found to be higher in hordeins fraction (His 2.24–2.69%; except BH-902, 1.49% and Phe 5.91–7.79%), followed by secalins (His 1.67–2.64% and Phe 5.67–7.09%) and gliadins (His 0.83–2.17% and Phe 5.19–6.48%) fractions. This implies that hordeins fraction might have relatively high propensity towards stabilizing the insoluble proteins through π- π or π-cation interactions with aromatic groups than secalin and gliadin fractions^50^. Thr and Val content was high in hordeins (Thr 3.01–3.87%; Val 4.38–6.01%;) and secalins (Thr 2.60–3.42%; Val 5.24–5.99%, except MCTLG-4, 6.87%) while low in gliadins (Thr 1.95–2.88%; Val 4.67–6.08%) fraction. The Ileu, Val and Leu which are mostly found in hydrophobic core and thus promote the folding process of protein more favourably^50^, these were found slightly higher in hordeins and secalins than gliadins fraction. The total essential amino acids (TEAA) were higher in hordeins (29.10–33.55%) and secalins (26.66–31.48%) while lower in gliadins (26.09–30.10%) fraction. Present results are in close proximity with the work conducted by Field et al^26^, who studied the amino acid content of prolamin fraction from wheat, rye and barley except for slightly lower value of phenylalanine (0.92–1.65%). Wang & Chen^14^ also reported similar observations on EAA of gliadins and hordeins fraction compared to our results. The use of thiolic compounds such as β-mercaptoethanol has been reported to prevent Trp degradation during acid hydrolysis to some extent^40^. Under the experimental conditions of moist heat, severe pressure, and this quantity of thiolic molecule, our findings on the usage of β-mercaptoethanol do not appear to prevent Trp degradation.

The non-essential amino acids (NEAA) are known to stabilize the protein polymeric structure through covalent, non-covalent, hydrophobic, electrostatic and van der waal interactions (Table 3, Figure S3). Gln + Glu were found to be the predominant NEAA with a mean value of 28.25–41.90% followed by Pro (8.67–14.97%). The estimated amount of Gln + Glu was 38.33–41.90% in gliadins, 35.14–39.37% in secalins and 38.35–30.93% in hordeins fraction. An opposite trend was obtained for Pro content in hordeins (10.09–14.97%), secalins (9.11–14.22%) and gliadins (8.67–13.66%) fractions. Experimentally the highest value of Gln + Glu was observed in gliadins fraction, HPW-155 (41.90%) and the lowest in hordeins fraction, BH-959 (28.25%), whereas the highest estimated amount of Pro was found in hordeins fraction from BH-902 (14.97%) and the lowest in gliadins fraction from HPW-155 (8.67%). This implies that the protein structure of gliadins is hypothesised to be stabilize by active participation of Glu in formation of H-bonding. As Pro lacks H-donor atom on its α-amino group, so act as β-sheet and/or α-helix breaker. Pro is mostly found in the end of α-helix, in turns and in loops. These results also correlated well to secondary structure data where gliadins are observed to be higher in β-sheet proportion while hordeins in α-helix structural component. Other NEAA were Ser (5.26–7.05%), Asn + Asp (2.69–5.56%), Tyr (2.81–4.93%), Arg (2.56–4.36%), Gly (2.19–4.02%) and Ala (2.00–3.72%). The Gln + Glu, Asn + Asp, Arg, Tyr, Pro and NEAA of prolamin fraction observed to vary significantly (p ≤ 0.05) among different cereals at inter and intra cultivar levels excluding Gln + Glu, Arg and Tyr among wheat cultivars. While Ser, Gly and Ala varied non-significantly (p ≥ 0.05) among different cereal and within their respective cultivars. The ordered structure of protein was strengthened by Ala and Glu (α-helix former) while weaken by Pro and Gly (α-helix or β-sheet breaker)^51^.

The Tyr residue was found to be higher in hordein (3.85–4.93%) followed by gliadin (3.35–3.83%) and secalin (2.81–3.27%) fractions, whereas Arg content was found to be the highest in hordeins (3.46–4.36%), followed by secalins (2.56–3.96%) and gliadins (2.87–3.74%) fractions. This means that in Tyr-Tyr covalent interactions in the secalin was comparatively less active than hordeins and gliadins fraction. Hou et al.^50^ suggested that the insolubilizing interactions has been facilitated by Asp, Glu, Arg and aromatic residues (Phe, Tyr, Trp, His). The role of Tyr-Tyr bond in stabilizing the toxic repetitive moiety of 33-mer gliadin was reported by Amundarain et al.^23^**.** The phenolic group of Tyr participates in formation of oxidative radical and thus stabilizes the intermediate proteins through the covalent bond of di-tyrosine.

Total non-essential amino acid (TNEAA) comprises of 66.45–73.91% of the total amino acid and being the highest in gliadins fraction from HPW-236 and the lowest from hordeins fraction from BH-959. The higher TNEAA of gliadins (69.90–73.91%) and secalins (68.52–73.34%) fractions are speculated to have more compact and stabilized protein–protein interaction as compared to hordeins (66.45–70.90%) fraction.

The NEAA profile obtained in present investigation is consistent with the literature. Similar results were reported by Field et al.^26^ for gliadin, secalin and hordein fractions except for slightly lower estimated amount of Asn + Asp (1.30–2.79%) and Arg (1.83–2.34%). Similarly, the estimated amount of NEAA in gliadins and hordeins fractions reported by Wang & Chen^14^ showed the similar findings except lower value of Asn + Asp (1.3–2.9%) and Arg (2.4–2.7%) while higher value of Pro (15.0–21.2%) content. The work of Šterna et al.^28^ on amino acid composition of hordein reported similar findings. Field et al.^26^ reported more serine and threonine in hordein than gliadin and secalin fractions which are similar to our finding, while ala was high in gliadin and secalin than hordein fraction. Our findings show non-significant (p ≥ 0.05) difference in the estimated amount of Ala in gliadin, secalin and hordein fraction. Gellrich et al.^27^ analysed secalin for amino acid and reported similar results except lower value for Arg, Lys and higher content of pro (20.4%) in comparison to current findings.

The International ImMunoGeneTics (IMGT) system of information categorizes the amino acids into three ‘hydropathy’ groups (Table 4) namely hydrophilic (Lys, Arg, Gln + Glu and Asn + Asp), hydrophobic (Phe, Trp, Ileu, Leu, Met, Cys, Ala, and Val) and neutral (Tyr, Pro, Gly, Thr, Ser and His)^52^. Out of total amino acids, the hydrophilic amino acids group constituted about 47.08–52.22% in gliadins, 45.28–47.97% in secalins and 41.12–45.09% in hordeins fraction. The highest hydrophilic amino acids content was observed in gliadins fraction from cv. HPW-155 and the lowest in hordeins fraction from cv BH-959. The hydrophilic amino acids of prolamin fraction were further sub categorized into basic and acidic amino acids, which accounted for 5.33–8.70% and 32.60–46.86% of the total amino acids respectively. The acidic AA residues which also include their amines (Gln + Glu and Asn + Asp) were found to be high in gliadins fraction (41.02–46.86%) and low in hordeins fraction (32.60–36.39%) whereas basic AA residues were high in hordeins fraction (8.11–8.70%) and low in gliadins fraction (5.33–6.56%). Acidic, basic and total hydrophillic amino acids of extracted prolamin fraction showed a significant (p ≤ 0.05) difference at intra and inter cultivar level.

The hydrophobic AA constituted 24.46–29.46% of the total amino acids and comprised of aliphatic, S-containing and some aromatic amino acids which accounted for 18.02–20.88%, 0.69–1.68% and 5.19–7.79% of the total amino acids respectively. The hydrophobic aromatic amino acids found to be higher in hordeins (5.91–7.79%) and secalins (5.68–7.09%) while lower in gliadins (5.19–6.48%) fraction. Prolamin aliphatic, S-containing and total hydrophobic AA group showed a non-significant (p ≥ 0.05) difference while the hydrophobic aromatic amino acids varied significant (p ≤ 0.05) at intra and inter cultivar level.

The total neutral amino acids of prolamin fraction varied from 22.21–30.48% and consisted of non-polar, polar and aromatic amino acids which accounted for 11.33–18.79%, 6.55–9.69%, and 2.81–4.93% respectively. The polar AAs were higher in hordeins (7.50–9.50%) and secalins (7.46–9.69%), while lower in gliadins (6.55–8.59%) fraction. Similarly, the non-polar AAs were higher in hordeins (14.07–18.79%) and secalins (12.53–16.92%), while lower in gliadins (11.33–15.96%) fraction. Hordeins fraction from BH-393 and gliadins fraction from HPW-236 accounted for the highest and the lowest proportion of polar amino acids respectively. Whereas the highest proportion of non-polar AA was observed in hordeins fraction cv BH-902 and the lowest in gliadins fraction cv HPW-155. The neutral aromatic AAs were found to be highest in hordeins fraction, BH-393 and the lowest in secalins fraction, MCTLG-3. This implies that Tyr-Tyr covalent interactions speculated to be more in hordein which attributed to greater aggregation tendency than gliadin and secalin fractions^23^.

The non-polar, polar, neutral aromatic and total neutral amino acids were observed to vary significantly (p ≤ 0.05) among prolamin fractions at inter and intra cultivar level except among wheat cultivars of neutral aromatic group where the values varied non-significantly (p ≥ 0.05). The variation in amino acid composition is considerably affected by genotype, irrigation practices, fertilizer application, and environmental conditions^53–58^.

### Secondary structure of prolamin fractions

**Figure 3:**
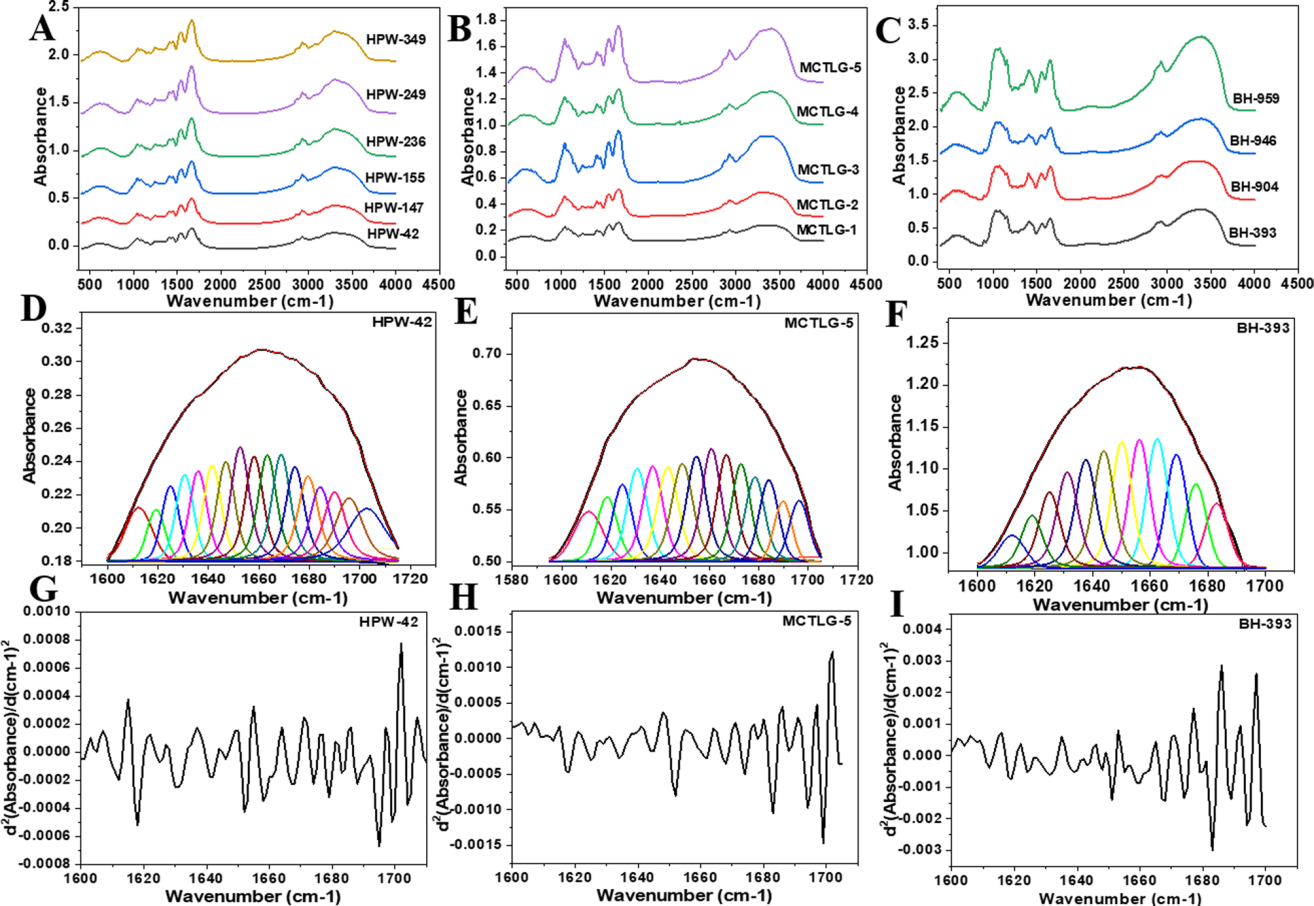
Original FTIR spectra of extracted prolamin (**A**) Gliadin, (**B**) Secalin, (**C**) Hordein; while Fourier-deconvoluted spectra in amide I region (1600–1700) of (**D**) HPW-42 Gliadin, (**E**) MCTLG-5 Secalin, (**F**) BH-393 Hordein; by confirming the peak positioned through their corresponding secondary derivative plot from (**G**) HPW-42 Gliadin, (**H**) MCTLG-5 Secalin and (**I**) BH-393 Hordein respectively.

The deconvoluted fitted curve and the relative amount of secondary structural components in amide I region (1600–1700) of extracted prolamin assigned to specific absorption frequencies are presented in Table S2, Fig. 3 and Figure S5. The structural component positioned between 1612–1624 cm^−1^, 1630–1642 cm^−1^, 1643–1648 cm^−1^, 1649–1659 cm^−1^, 1660–1685 cm^−1^ and 1686–1699 cm^−1^ has been assigned to inter-molecular β-sheet, β-sheet, random coil, α-helix, β-turn and β-turn + β-sheet secondary structure respectively^29,41,46,59^.

The regions centred at 1699 cm^−1^, 1695 cm^−1^ and 1688 cm^−1^ were assigned as β-turn + β-sheet of prolamin and found higher in gliadins (11.90–18.55%) followed by secalins (6.72–12.33%) and least in hordeins (3.39–5.23%) fraction. The dominant band position assigned for β-turn observed in gliadins (1683 cm^−1^, 1679 cm^−1^, 1673 cm^−1^, 1668 cm^−1^, 1663 cm^−1^ and 1661 cm^−1^), secalins (1684 cm^−1^,1679 cm^−1^, 1675 cm^−1^, 1671 cm^−1^, 1668 cm^−1^ and 1665 cm^−1^) and hordeins (1683 cm^−1^,1679 cm^−1^, 1674 cm^−1^, 1671 cm^−1^, 1668 cm^−1^ and 1663 cm^−1^) was found in the range of 26.32–37.91%. Indeed, the higher proportion of β-turn in prolamin fraction was supposed to be attributed due to higher content of proline in repetitive domain of prolamin^45^. Wouters et al.^46^ suggested that β-turn are rich in ω-gliadin and possess only lower amount of β-sheet and α-helix, which seems consistent with our findings. The ω-gliadin and C-hordein were also found also rich in β-turn as revealed by Purcell et al.^15^.

The dominant signature region in gliadins (1652 cm^−1^ and 1656 cm^−1^), secalins (1654 cm^−1^ and 1658 cm^−1^) and hordeins (1651 cm^−1^, 1655 and 1658 cm^−1^) was assigned to α-helix. This structural conformation is generally attributed to non-repetitive domain of gliadin^45^. The relative amount of α-helix was found to be higher in hordeins (20.32–28.95%), followed by secalins (13.81–19.35%) and gliadins (15.06–17.74%) fractions. Wouters et al.^46^ also revealed that the α and γ-gliadin are relatively richer in β-turn and α-helix structural components. The relative proportion of random coil component around 1648 cm^−1^ (secalins and hordeins) and 1644 cm^−1^ (hordeins) was found higher in hordeins (9.05–10.28%), followed by secalins (7.29–8.33%) whereas absent in gliadins fraction. Gliadin exhibited a dominant β-sheet structural component around 1639 cm^−1^, 1634 cm^−1^, 1630 cm^−1^ corresponding to 20.33–26.19%, while secalins (1637 cm^−1^ and 1630 cm^−1^) accounted for 19.06–22.56% and hordeins (1639 cm^−1^ and 1632 cm^−1^) to 15.78–18.81% of its total amide I region. The bands centred at 1624 cm^−1^, 1618 cm^−1^ and 1612 cm^−1^ originating from intermolecular β-sheet secondary structure with stronger hydrogen bond were found to be higher in secalins (12.35–17.41%), followed by gliadins (11.22–14.73%) and hordeins (9.72–15.30%). Mangavel et al.^45^ revealed the combined contribution of 1622 cm^−1^ and 1693 cm^−1^ bands to antiparallel intramolecular β-sheet which is a characteristic feature of protein aggregation. Significant difference (p ≤ 0.05) existed between secondary components of extracted prolamin at inter and intra cultivar level.

The previous report on the secondary structure of hydrated gluten via FTIR showed absence of random coil component^41^ similar to our findings in the present work. Our results were also in consistent with that of Li et al.^60^, who reported more β-turn and relatively lesser amount of β-sheet structural configuration in soluble glutenin and gliadin protein fractions. Findings on the structural properties of gliadin containing β-sheet (39.46–40.18%), random coil (13.73–14.24%), α-helix (13.98–14.23%) and β-turn (31.62–32.46%) were in close agreement with our results^61^.

### Particle characteristics

The prolamins exist as three-dimension structured particle and its microstructure was analysed using analytical instruments to elucidate its properties.

#### Dynamic light scattering (DLS)

The prolamin fractions were analysed for particle size distribution (PSD) in terms of polydispersity index (PDI) and hydrodynamic diameter (D_h_) in solution of acetonitrile:water:formic acid:: 50:50:0.1 under our experimental condition as presented in Table S3, Figure S6. PDI values for prolamins fall in the range 0.49–0.87. Lower PDI value *i.e.* less than 0.5 indicates more uniform PSD and existence of monodisperse system^62^. In the present study, PDI of the secalins (0.66–0.87), hordeins (0.58–0.83) and gliadins (0.49–0.64) were higher indicating disparity in particle size.

The gliadins and hordeins mainly existed in monomeric form with hydrodynamic diameter (D_h_) in the ranges between 1.23–1.83 nm and 6.67–7.62 nm respectively. The hordeins from BH-946 (7.62 nm) had higher and gliadins from HPW-42 (1.23 nm) had lower hydrodynamic diameter. Gliadins showed monomeric units nonetheless hordeins shows the existence of some higher ordered aggregates. This fact is also supported by amino acid composition that the higher proportion of basic AA and S-containing AA residues in hordeins show their tendency in formation of aggregates through strong electrostatic and hydrostatic interaction^63^.

The secalins showed a bimodal PSD with two populations in the solution. The particle size of small and large sized population varied from 7.73–18.44 nm and 26.48–68.33 nm, whereas, their proportion ranged from 99.60–99.90% and 0.10–0.40% respectively. It indicated that the small sized particles were abundant in number while large sized particle were scanty. The large sized particles were observed in secalin fraction from MCTLG-3 (18.44 nm; 68.33 nm) and the small sized from MCTLG-4 (7.73 nm; 26.48 nm) in peak 1 and 2 respectively. The PDI and D_h_ of the investigated prolamin have been found to vary significantly (p ≤ 0.05) among different extracted prolamin samples including intra-cultivar differences except wheat cultivars which shares non-significant differences (p ≥ 0.05)**.**

The zeta potential (ZP) profile of the gliadins, secalins and hordeins fraction observed a positive value in the range of 23.53–27.00 mV, 11.23–16.60 mV and 4.10–7.98 mV respectively. The ZP values were found to be significantly (p ≤ 0.05) different at inter as well as intra-cultivar levels. The relatively higher zeta potential values of gliadin fraction indicated the high stability of their monomeric protein subunits. Gliadins and hordeins fractions had a monomodal PSD while secalins fraction had a bimodal PSD by number-based size distribution (Figure S6a-d). The secalin fraction have relatively lower ZP than gliadins fraction and these lower ZP values are very well corroborated by the size measurement values of the secalins fraction where large size particles have been observed along with small particle that results in its bimodal PSD. The lower zeta potential values (< 30 mV) indicate the lower electrostatic charge on the particle hence lower repulsions among them that can leads to the aggregation of particles to form bigger aggregates hence the presence of large size particles in size distribution profile of secalins fraction can be correlated with the its lower ZP values.

Further the intensity-based size distribution (Figure S6e–g) showed higher ordered aggregates in hordein followed by secalins and gliadins. On the other hand, the substantially lower ZP values for hordeins fraction reflected the greater tendency of protein motifs to form aggregates due to lower surface charge. The constituent protein units predominantly exist in higher ordered form in secalins and hordeins fraction compared to gliadins fraction, which was correlated by their corresponding PDI value. The low ZP in case of hordein fraction is probably due to its high basic amino acid contents which might be involved in π-interaction and increase aggregation propensities^50^.

Zavareze et al.^64^ has reported that zeta-potential of gliadin was about 30 mV and had remarkable colloidal stability. However, gliadin nano-particles prepared by controlled aggregation had D_h_, PDI and ZP of 190–220.6 nm, 0.067–0.232 and 14.8–18.3 mV respectively^46^. Peng et al.^65^ have also demonstrated the high zeta potential which indicated greater electrostatic repulsions among the protein molecules and poses higher electrostatic barrier which prevents protein aggregation. The overall zeta-potential of gliadins, secalins and hordeins was observed to be in decreasing order. It further indicated the solution stability of these proteins was also in the same order. The higher colloidal stability of gliadins suspension is attributed to the high glutamine content which is engaged in hydrogen bonding with water^21^. The variation in zeta potential between inter and intra cultivar of present and earlier studies might be due to difference of dispersion media, amino acid side chains, pH, ionic strength of solution and temperature during extraction/analysis process^21,64^.

#### SEM and EDX

**Figure 4:**
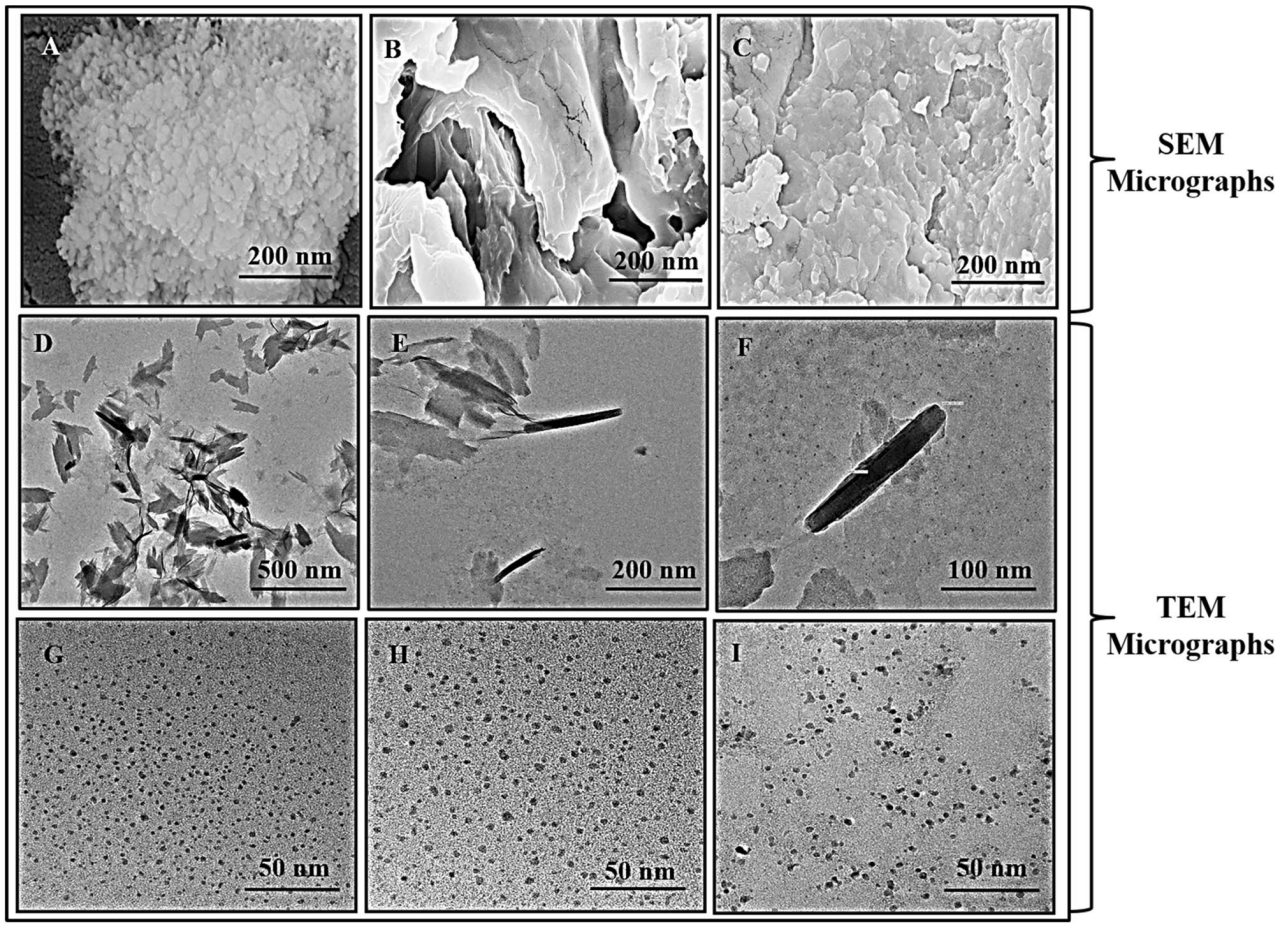
SEM micrographs showing surface morphology (**A**) Gliadin, HPW-42, (**B**) Secalin, MCTLG-5, (**C**) Hordein, BH-393. TEM micrographs showing (**D**) rod shaped HPW-42 Gliadin at 500 nm, (**E**) rod shaped HPW-42 Gliadin at 200 nm (**F**) rod shaped HPW-42Gliadin at 100 nm, (**G**) HPW-42 Gliadin at 50 nm, (**H**) MCTLG-5Secalin at 50 nm, and (**I**) BH-393Hordein at 50 nm.

Scanning Electron Microscopy (SEM) and Energy Dispersive X-Ray Analyzer (EDX) were used to investigate the surface morphology and elemental composition of prolamin respectively as elucidated in Fig. 4, Figure S8 and Table S4. Furthermore, the cultivar HPW-42 gliadin, MCTLG-5 secalin and BH-393 hordein were selected for further morphological, internal structural and elemental analysis, based on relatively higher proportion of LMW group (α/β, γ- gliadin and LMW-GS) and lower proportion of HMW group at intra cultivar level. Morphology by SEM illustrated the globular particle arrangement of gliadin while sheet-like and stacked flaky structure was observed for secalin and hordein respectively. Similar globular particle arrangement has been reported in gliadin nanoparticles^5^ which seems consistent with our findings.

EDX analysis is an extended application of SEM technology which reveals the element composition of matter. Here the EDX data showed the presence of important elements in the prolamins. The EDX analysis revealed the major elements present in the prolamin fractions as C (52.33–57.60%), O (27.03–35.26%) and N (10.43–14.20%), while minor elements as S (0.12–0.26%), P (0.37–0.61%), Na (0.28–0.75%) and I (0.25–0.54%) expressed in atomic percentage. The atomic percentage is considered when the stoichiometry arrangement of elements is important. Similarly, the EDX analysis expressed in weight percentage showed the major elements as C (43.16–50.71%), O (30.99–38.94%) and N (9.46–14.80%), while minor elements as S (0.25–0.63%), P (0.84–1.31%), Na (0.42–1.18%) and I (1.97–4.56%). The weight percentage is considered when contribution of each element to the total weight of the molecule is important. The EDX analysis suggested that the gliadin fraction consisted of C, N, O with traces of S and P, Na and I were detected possibly due to NaI salt used in the extraction procedure. This implies that the relative ratio of C:O:N:S (weight percentage) in prolamin followed the ascending order, being higher in hordein (113:156:43:1), followed by secalin (110:76:21:1) and gliadin (77:48:24:1). It was noted that O is two times the amount of N in gliadin fraction while 3.6 times in secalin and hordein fractions.

#### X-ray diffraction (XRD)

**Figure 5:**
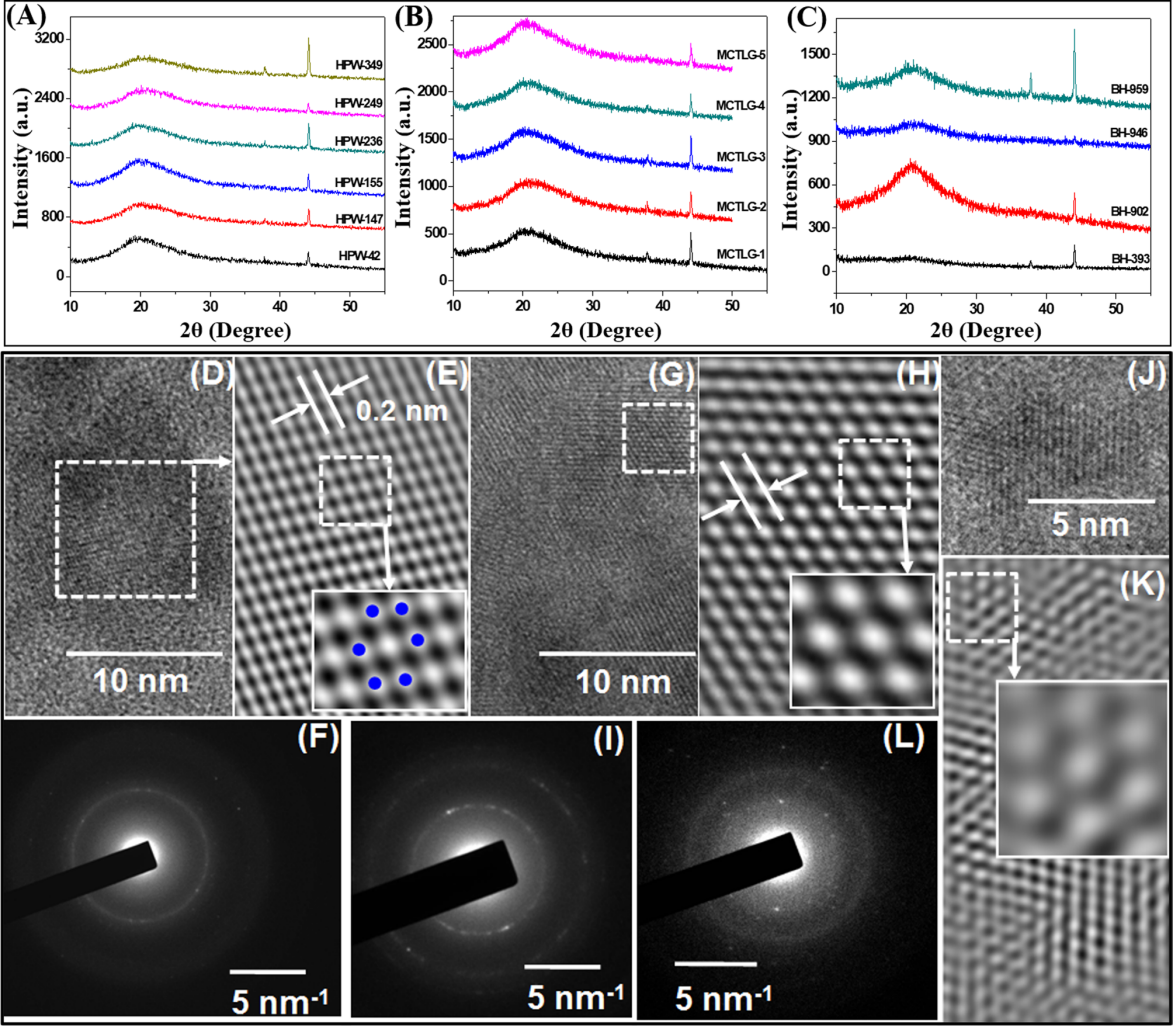
X-ray diffractograms of (**A**) Gliadin, (**B**) Secalin and (**C**) Hordein. HRTEM image of (**D**) HPW-42 Gliadin, (**E**) is digitally filtered image of area highlighted in (**D**). (**G**,**H**) correspond to Secalin, MCTLG-5 and (**I**) and (**K**) correspond to Hordein, BH-393. (**F**,**I**,**L**) are respective selected area electron diffraction pattern from Gliadin, Secalin and Hordein. These images were taken from respective HRTEM image area shown in (**A**,**G**,**I**). Note that in all images digitally filtered images are produced from highlighted area in respective HRTEM images as shown in Figure above.

XRD is the primary and most widely employed technique to examine the micro-structural properties of various compounds. The XRD patterns of different prolamin fractions are presented in Fig. 5a–c & S7. A broad hump at around 20° indicated the amorphous nature of all the samples which was also reported by Jia et al.^20^ in gliadin. High Glu and Pro content was observed in prolamin fractions and significant contamination of extracted prolamin with albumin + globulin which might have contributed to this amorphous structure^66^. The outcome of Ebrahimi et al.^67^ also supported this finding and showed the presence of broad hump around 20° in XRD pattern of pure BSA, but crystalline peaks were not observed in 5–40^o^ diffraction 2θ scan. Prolamin fractions from different cultivars showed diffraction peaks at 2θ values of 10.4°, 37.8° and 44.1° which indicated the existence of a regular periodic arrangement of molecules or crystallinity.

The size of the crystallites corresponding to above mentioned diffraction peaks was determined using Scherrer's formula (Eq. 4). The crystallite size corresponding to 44.1° diffraction peaks in gliadins, secalins and hordeins fraction varied from 12.29–13.67 nm, 12.47–13.05 nm and 12.29 –14.13 nm respectively (Table S5). The relatively smaller crystallite size at 37.8° diffraction peak was observed in gliadins (11.23–14.33 nm) followed by hordeins (13.40–16.63 nm) and secalins (14.01 –17.31 nm) fractions. Variable sizes were observed at 10.4^o^ diffraction peak in gliadins (13.05–17.94 nm), hordeins (16.79–17.94 nm) and secalins (15.47–18.35 nm) fractions. The regular arrangement can be classified into Category I—comprises of three organized patterns corresponding to 10.4°, 37.8° and 44.1° diffraction peaks and Category II – comprises of two organised patterns corresponding to 10.4^o^ and 44.1°. The pattern of category I exists in most of the prolamins, whereas category II was found in small number of prolamin fractions i.e., gliadins (HPW-42, HPW-155, HPW-249) and hordeins (BH-946) fractions. Jia et al. ^20^ also reported crystalline peaks in XRD analysis of gliadin, however these peaks were at different position. Similar crystalline diffraction was reported by Guan et al.^30^ for electrospun fibre of hordein at 2θ values of 16.88°, 38.3° and 44.6°, which was in close proximity to our findings.

The inter-planar spacing, ’d’, was calculated from the peak positions using Bragg's equation (Table S6). The inter-planar spacing corresponding to the diffraction peak at 44.1^o^ was 0.21 nm, 37.8^o^ was 0.24 nm and 10.4^o^ was 0.85 nm. The report of Guan et al.^30^ revealed similar inter-planar distance for electrospun fibre of hordein to the diffraction peak of 16.88°, 38.3° and 44.6^o^ as 0.52 nm, 0.23 nm and 0.20 nm respectively.

#### Transmission electron microscopy

Transmission Electron Microscopy was used to study the morphology and nanostructure of prolamin fractions. The micrograph was analysed for surface morphology (Fig. 4D–I), lattice planes and ring diffraction patterns of selected area (Fig. 5D–L). Morphology of gliadins, secalins and hordeins fraction showed a compact spherical structure (Fig. 4G–I) which might be due to active participation of *α*/*β*-gliadins polypeptides^68^. A rod like structure (Fig. 4 D-F) was also observed in gliadins with an average diameter of 17.94 nm and length of 250 nm. Formation of such structures is attributed to involvement of ω and γ-gliadin polypeptides^68^. Ang et al.^17^ also supported the existence of two structures of gliadins as compact globular and rod-shaped. The histogram shows the particle size distribution of compact globular structures in prolamin fractions and rod structure of gliadins fraction (Figure S9). The average particle size was found to be smallest for gliadins (3.88 nm), followed by hordeins (4.32 nm) and largest in secalins (5.79 nm) fraction.

Figure 5 (D-L) display transmission electron microscopy images acquired on three different samples. Panel (D) represents HR-TEM image from gliadin. Panel E is corresponding digitally filtered image from highlighted area shown in D. One can clearly see hexagonal symmetry and presence of lattice fringes with a spacing of 0.2 nm corresponding to (Fig. 5E) planes of gliadin, HPW-42. Panel (F) represents the corresponding SAED pattern from the area shown in panel (A). A ring pattern clearly indicates the polycrystalline nature of the sample. Corresponding inter-planar spacing is found to be 0.21 nm, matching well with panel (E). Panels (G) and (H) correspond to secalin, MCTLG-5 and clearly indicate hexagonal symmetry (inset) and presence of planes with inter-planar spacing of 0.33 nm. Panel (I) represents corresponding SAED image showing two rings with an inter-planar spacing of 0.21 and 0.34 nm. Similar images for hordein, BH-393 are shown in panels (J)-(L). Where, (J) is HR-TEM image and (K) is corresponding noise filtered image from area shown in (J) and also display fringes with a spacing of 0.35 nm. The inset in panel (K) also displays hexagonal symmetry of the sample. This space saving molecular arrangement might be responsible for gliadin to act as plasticizer. Similar molecular arrangement of gliadin as hexagonal close packed was previously reported by Rasheed et al.^19^ which is in line with our findings.

It is to be noted that there is a lack of detailed XRD analysis and corresponding (hkl) values for different planes present in these sample. A few recent reports that appeared on same sample also lack the detailed XRD analysis and hence accurate Miller indices cannot be assigned to these planes^18–20^.

### Comparison among analytical techniques

Particle size of the prolamins (Table 5) observed for gliadins (1.12–6.06 nm), secalins (3.23–9.47 nm) and hordeins (2.13–6.83 nm) fractions using TEM whereas for gliadins (1.23–1.83 nm), secalins (6.45–11.96 nm) and hordeins (6.67–7.62 nm) fractions using DLS and for gliadins (44.1°, 12.29–13.67 nm; 37.8°, 11.23–14.33 nm), secalins (44.1°, 12.47–13.05 nm; 37.8°, 14.01 –17.31 nm) and hordeins (44.1°, 12.29 –14.13 nm; 37.8°, 13.40–16.63 nm) fractions using XRD. Among the prolamin fractions, the particle size of secalins was the largest, followed by hordeins and gliadins fraction. Among the analytical techniques, largest particle size was observed on XRD while TEM and DLS revealed particle size in a close range. However, the particle size range overlapped in all the three prolamin fractions.

**Table 5:** The comparative particle size of gliadin, secalin and hordein obtained from XRD, DLS and TEM analysis.

Gliadin	XRD Crystallite Size (nm)	DLS Hydrodynamic Diameter D_h_ (nm)	TEM Particle Size (nm)	Secalin	XRD Crystallite Size (nm)	DLS Hydrodynamic Diameter D_h_ (nm)	TEM Particle Size (nm)	Hordein	XRD Crystallite Size (nm)	DLS Hydrodynamic Diameter D_h_ (nm)	TEM Particle Size (nm)
HPW-42	12.66	1.23 ± 0.38	3.88 ± 0.80	**MCTLG-1**	12.66	11.96 ± 1.58		BH-393	12.29	6.94 ± 0.67	5.79 ± 1.13
	17.94				14.84	39.82 ± 3.53			14.08		
HPW-147	11.23	1.39 ± .17	–		16.79				17.16		
	13.05			**MCTLG-2**	12.47	10.35 ± 1.05		BH-902	13.05	6.67 ± 1.66	–
	13.54				15.68	39.90 ± 4.05			16.63		
HPW-155	12.29	1.71 ± 0.13	–		15.47				17.94		
	16.11			**MCTLG-3**	12.66	18.44 ± 4.33		BH-946	14.13	7.62 ± 0.58	–
HPW-236	13.67	1.58 ± 0.09	–		14.33	68.33 ± 6.58			16.79		
	14.33				17.54			BH-959	12.47	7.03 ± 1.09	–
	17.94			**MCTLG-4**	13.05	7.73 ± 1.79			13.4		
HPW-249	12.47	1.65 ± 0.10	–		14.01	26.48 ± 9.83			17.54		
	17.61				16.79						
HPW-349	12.29	1.83 ± 0.13	–	**MCTLG-5**	12.85	8.75 ± 2.08	4.32 ± 0.82				
	13.62				17.31	27.41 ± 10.62					
	15.78				18.35						

The crystalline microstructure of prolamin was shown by both XRD and TEM studies. SEM images and low resolution TEM images confirmed the morphological differences between different samples. Further, HR-TEM images from samples indicated the presence of variable sized nanostructures.

The d-spacing was estimated from HR-TEM lattice plane pattern, Selected Area Electron Diffraction (SAED) pattern and XRD data. The d-spacing (Table S6) observed by HR-TEM, SAED and XRD for gliadins fraction was 0.21 nm, 0.29 nm and 0.21–0.85 nm whereas for hordeins fraction, it was 0.35 nm, 0.32 nm and 0.21–0.85 nm while for secalins fraction, it was 0.32 nm, 0.21–0.34 nm and 0.21–0.85 nm, respectively. For gliadins fraction the d-spacing obtained via XRD and HR-TEM imaging matches with each other. Further, as evident from SAED pattern and XRD data, all three samples have polycrystalline nature. These periodic regular arrangements probably attributed to the subsistence of β-sheet structure among the prolamin and disulphide bonding as a result of intra-molecular interaction which mainly involves cysteine residues in polypeptide chains^69^. Markgren et al.^63^ highlighted the role of cysteine amino acid in intra-molecular disulphide bonding, and stated that the intra-molecular interaction enhances the tendency of formation of more ordered β-sheet configuration. Similarly, Jung et al.^70^ also reported the crystalline structure of proteins by XRD studies and attributed it to the β-sheet structure**.** Rasheed et al.^19^ have also reported that the modified gliadin with nano-crystalline structure consists of high proportion of β-sheet structure along with irreversible linkages such as covalent disulphide bonds.

## Conclusion

Freeze dried powder of gliadins, secalins and hordeins fraction had a light cream color. The crude protein content of extracted gliadins was found to be higher than secalins and hordeins fraction. The secondary structure of extracted prolamin revealed higher α-helix and random coil components in hordeins, while higher β-sheet and β-turn components in gliadins. Prolamin fractions were abundant in Gln + Glu acid and Pro content among the non-essential amino acids while Leu, Phe and Val among the essential amino acids. SDS-PAGE revealed that prolamin fractions contained minor amount of HMW-GS while cross-contamination with significant amount of albumin and globulin. This method seems to extract majority of ω-prolamin fractions and had well resolved LMW region, but still needs stepwise extraction with sodium chloride and water so that the prolamin fractions are free from these metabolic active proteins. Furthermore, the instrumental analysis reported here might be influenced by presence of albumin and globulin fractions. SEM images showed globular morphology of gliadin fraction while sheet like or stacked flaky morphology of hordein and secalin fractions. TEM analysis of gliadin fraction showed a compact spherical structure as well as a rod like structure. Particle size determined by TEM and DLS were in close proximity. XRD results indicated that prolamin had both amorphous as well as crystalline structure. Furthermore TEM-SAED pattern also revealed the polycrystalline nature and a hexagonal symmetry. The zeta potential of the gliadin fraction was higher followed by secalin and hordein fractions. The energy dispersive X-ray analyzer revealed the major elements present in the prolamin fractions as C, O and N, while minor elements as S, P, Na and I.

## Supplementary Information

Supplementary Information.
