# Supplementary material for: Characterization of gliadin, secalin and hordein fractions using analytical techniques
Source: Sci Rep. 2021 Nov 30;11:23135. doi: 10.1038/s41598-021-02099-0 (PMC8633357; doi:10.1038/s41598-021-02099-0)
Supplement: Supplementary file 1 — Supplementary Information. [file 41598_2021_2099_MOESM1_ESM.docx]

**Characterization of gliadin, secalin and hordein fractions using analytical techniques.**

Monika Rani^1^, Dalbir Singh Sogi^1^*, Balmeet Singh Gill^1^

^1^*Department of Food Science and Technology, Guru Nanak Dev University, Amritsar, Punjab, India.*

*Corresponding author: Email-address: [sogids@gmail.com](mailto:sogids@gmail.com)***,*** FAX: +91-0183-2258820, (***Dalbir Singh Sogi)***.

**Supporting Information**

**Table S1.** Protein content, water activity and Hunter color Lab (CIE) of Gliadin, Secalin and Hordein fractions of different cultivars.

Mean±SD with different superscripts in column differ significantly (*p* ≤ 0.05); *n* = 3 for each treatment

| **Cereal Cultivar** | **Protein content (%)** | **Water activity** | **L*** | **a*** | **b*** | **Chroma** | **Hue angle** |
| --- | --- | --- | --- | --- | --- | --- | --- |
| ***Gliadin*** | | | | | | | |
| HPW-42 | 69.19±3.69^AB^ | 0.73±0.00^C^ | 63.71±3.69^AB^ | 1.87±0.41^ABC^ | 13.93±2.23^A^ | 14.05±2.27^A^ | 82.40±0.47^BCDE^ |
| HPW-147 | 64.94±6.65^ABCD^ | 0.72±0.00^C^ | 63.94±2.99^AB^ | 1.60±0.23^ABCD^ | 11.85±1.69^AB^ | 11.96±1.70^AB^ | 82.28±0.66^CDE^ |
| HPW-155 | 68.13±3.69^AB^ | 0.73±0.01^C^ | 65.08±3.93^AB^ | 1.53±0.30^ABCDE^ | 11.72±1.58^AB^ | 11.82±1.59^AB^ | 82.56±1.24^BCDE^ |
| HPW-236 | 67.07±5.53^ABC^ | 0.71±0.00^CD^ | 61.16±0.55^BC^ | 2.06±0.51^AB^ | 11.39±0.58^AB^ | 11.58±0.66^AB^ | 79.87±2.02^EF^ |
| HPW-249 | 68.13±3.69^AB^ | 0.79±0.01^B^ | 66.25±3.06^AB^ | 1.77±0.24^ABC^ | 13.37±1.11^A^ | 13.49±1.06^A^ | 82.36±1.72^BCDE^ |
| HPW-349 | 70.26±5.53^A^ | 0.73±0.00^C^ | 64.81±3.35^AB^ | 1.72±0.49^ABC^ | 9.68±1.15^BCD^ | 9.84±1.22^BCD^ | 80.06±1.74^DEF^ |
| ***Secalin*** | | | | | | | |
| MCTLG-1 | 49.66±7.48^BCDEF^ | 0.80±0.01^AB^ | 53.13±0.78^D^ | 0.81±0.09^EF^ | 6.90±0.43^D^ | 6.95±0.43^D^ | 83.32±0.34^BC^ |
| MCTLG-2 | 59.98±9.82^ABCDE^ | 0.82±0.01^A^ | 55.08±0.07^CD^ | 0.56±0.08^F^ | 7.16±0.21^CD^ | 7.18±0.21^D^ | 85.55±0.51^AB^ |
| MCTLG-3 | 60.70±3.75^ABCDE^ | 0.80±0.00^AB^ | 56.07±0.68^CD^ | 1.22±0.05^BCDEF^ | 9.20±0.21^BCD^ | 9.28±0.21^BCD^ | 82.46±0.21^BCDE^ |
| MCTLG-4 | 60.22±7.45^ABCDE^ | 0.80±0.01^AB^ | 53.57±1.28^D^ | 1.54±0.13^ABCDE^ | 9.25±0.53^BCD^ | 9.37±0.55^BCD^ | 80.53±0.46^CDEF^ |
| MCTLG-5 | 47.50±9.89^CDEF^ | 0.79±0.00^B^ | 54.56±1.39^CD^ | 2.06±0.13^A^ | 10.13±0.72^BC^ | 10.33±0.73^BC^ | 78.51±0.24^F^ |
| ***Hordein*** | | | | | | | |
| BH-393 | 40.86±9.86^EF^ | 0.67±0.01^EF^ | 60.49±2.03^BC^ | 0.95±0.08^DEF^ | 8.21±0.25^CD^ | 8.26±0.25^BCD^ | 83.39±0.53^BC^ |
| BH-902 | 34.69±3.75^F^ | 0.66±0.01^F^ | 68.12±1.27^A^ | 0.89±0.02^DEF^ | 7.41±0.41^CD^ | 7.46±0.41^BCD^ | 83.17±0.27^BCD^ |
| BH-946 | 45.17±6.45^DEF^ | 0.67±0.01^EF^ | 64.72±1.91^AB^ | 1.28±0.21^BCDEF^ | 8.18±0.32^CD^ | 8.28±0.29^BCD^ | 81.06±1.70^CDEF^ |
| BH-959 | 34.06±6.81^F^ | 0.69±0.01^DE^ | 63.52±1.76^AB^ | 0.53±0.12^F^ | 9.61±0.12^BCD^ | 9.63±0.12^BCD^ | 86.85±0.76^A^ |

**Table S2:** The relative area (%) of the Fourier-deconvoluted bands of the extracted prolamin corresponds to components of secondary structure.

Mean±SD with different superscripts in column differ significantly (*p* ≤ 0.05); *n* = 3 for each treatment.

| **Prolamin** | **Intermolecular β-sheet** | **β-sheet** | **Random coil** | **α-helix** | **β-turn** | **β-turn+β-sheet** |
| --- | --- | --- | --- | --- | --- | --- |
| ***Gliadin*** | | | | | | |
| HPW-42 | 11.22±0.20^EF^ | 21.56±1.45^BCDE^ | 0.00±0.00^E^ | 15.22±0.68^EF^ | 33.46±1.08^BC^ | 18.55±0.47^A^ |
| HPW-147 | 11.32±0.48^EF^ | 25.93±0.33^A^ | 0.00±0.00^E^ | 15.38±0.26^EF^ | 30.61±0.64^CD^ | 16.77±0.15^AB^ |
| HPW-155 | 11.22±0.51^EF^ | 26.19±0.98^A^ | 0.00±0.00^E^ | 15.06±0.52^EF^ | 35.63±0.20^AB^ | 11.90±0.22^D^ |
| HPW-236 | 12.32±0.22^CDEF^ | 23.66±0.20^AB^ | 0.00±0.00^E^ | 17.74±1.07^CDE^ | 30.36±1.06^CD^ | 15.92±0.59^AB^ |
| HPW-249 | 12.39±0.51^CDE^ | 20.33±0.98^BCDEF^ | 0.00±0.00^E^ | 16.64±0.52^DEF^ | 37.91±0.20^A^ | 12.72±0.22^CD^ |
| HPW-349 | 14.73±0.45^BC^ | 21.09±0.37^BCDE^ | 0.00±0.00^E^ | 16.91±0.84^DEF^ | 32.11±1.26^BC^ | 15.17±0.49^BC^ |
| ***Secalin*** | | | | | | |
| MCTLG-1 | 12.35±1.17^CDEF^ | 22.56±0.29^BC^ | 7.95±0.74^CD^ | 13.81±1.86^F^ | 31.00±0.76^CD^ | 12.33±1.15^D^ |
| MCTLG-2 | 17.41±1.90^A^ | 19.06±1.95^DEFGH^ | 8.33±0.77^BCD^ | 14.91±0.60^EF^ | 31.86±0.65^C^ | 8.42±0.69^EF^ |
| MCTLG-3 | 15.98±0.57^AB^ | 19.77±1.00^CDEFG^ | 7.99±0.83^CD^ | 19.35±2.18^CD^ | 27.98±2.18^DE^ | 8.93±0.89^EF^ |
| MCTLG-4 | 15.79±0.82^AB^ | 20.03±1.79^CDEFG^ | 7.29±0.08^CD^ | 15.32±0.60^EF^ | 31.47±1.81^CD^ | 10.10±0.75^DE^ |
| MCTLG-5 | 16.25±0.97^AB^ | 22.31±0.61^BCD^ | 7.98±0.35^CD^ | 16.57±0.83^DEF^ | 30.17±1.96^CD^ | 6.72±0.96^FG^ |
| ***Hordein*** | | | | | | |
| BH-393 | 15.30±0.32^AB^ | 16.94±1.43^GH^ | 9.10±0.33^ABC^ | 28.95±0.65^A^ | 26.32±0.99^E^ | 3.39±1.29^H^ |
| BH-902 | 9.72±0.44^F^ | 15.78±0.38^H^ | 9.05±0.20^ABC^ | 28.86±0.99^A^ | 32.36±1.71^BC^ | 4.23±0.27^GH^ |
| BH-946 | 12.05±0.46^DEF^ | 17.64±1.18^FGH^ | 10.28±1.48^A^ | 22.92±0.94^B^ | 32.26±0.81^BC^ | 4.84±1.66^GH^ |
| BH-959 | 14.23±1.77^BCD^ | 18.81±1.64^EFGH^ | 9.93±0.40^AB^ | 20.32±0.87^BC^ | 31.48±0.89^CDA^ | 5.23±0.59^GH^ |

**Table S3. Polydispersity index, hydrodynamic diameter and zeta potential of freeze-dried gliadin, secalin and hordein fractions.**

Mean±SD with different superscripts in column differ significantly (*p* ≤ 0.05); *n* = 3 for each treatment

| **Cereal Prolamin** | **Polydispersity Index** | **Hydrodynamic diameter D_h_ (nm)** | | **Number [%]** | | **Zeta Potential (mV)** |
| --- | --- | --- | --- | --- | --- | --- |
|  |  | **Peak 1** | **Peak 2** | **Peak 1** | **Peak 2** |  |
| ***Gliadin*** | | | | | | |
| HPW-42 | 0.51±0.11^A^ | 1.23±0.38^D^ | - | 100.00±0.00^A^ | - | 23.53±0.46^B^ |
| HPW-147 | 0.59±0.06^A^ | 1.39±.17^D^ | - | 100.00±0.00^A^ | - | 27.00±0.96^A^ |
| HPW-155 | 0.49±0.04^A^ | 1.71±0.13^D^ | - | 100.00±0.00^A^ | - | 25.53±0.12^AB^ |
| HPW-236 | 0.64±0.14^A^ | 1.58±0.09^D^ | - | 100.00±0.00^A^ | - | 25.93±0.35^A^ |
| HPW-249 | 0.61±0.18^A^ | 1.65±0.10^D^ | - | 100.00±0.00^A^ | - | 26.10±0.44^A^ |
| HPW-349 | 0.57±0.10^A^ | 1.83±0.13^D^ | - | 100.00±0.00^A^ | - | 25.10±1.08^AB^ |
| ***Secalin*** | | | | | | |
| MCTLG-1 | 0.67±0.42^A^ | 11.96±1.58^B^ | 39.82±3.53^B^ | 99.83±0.06^A^ | 0.17±0.06^A^ | 14.00±0.82^D^ |
| MCTLG-2 | 0.87±0.12^A^ | 10.35±1.05^BC^ | 39.90±4.05^B^ | 99.90±0.00^A^ | 0.10±0.00^A^ | 16.60±0.72^C^ |
| MCTLG-3 | 0.74±0.05^A^ | 18.44±4.33^A^ | 68.33±6.58^A^ | 99.90±0.00^A^ | 0.10±0.00^A^ | 13.90±0.53^D^ |
| MCTLG-4 | 0.66±0.33^A^ | 7.73±1.79^BC^ | 26.48±9.83^C^ | 99.63±0.38^A^ | 0.37±0.38^A^ | 15.03±1.42^CD^ |
| MCTLG-5 | 0.66±0.06^A^ | 8.75±2.08^BC^ | 27.41±10.62^BC^ | 99.60±0.52^A^ | 0.40±0.52^A^ | 11.23±1.46^E^ |
| ***Hordein*** | | | | | | |
| BH-393 | 0.83±0.08^A^ | 6.94±0.67^C^ | - | 100.00±0.00^A^ | - | 7.98±0.59^F^ |
| BH-902 | 0.61±0.10^A^ | 6.67±1.66^C^ | - | 100.00±0.00^A^ | - | 4.68±0.66^G^ |
| BH-946 | 0.58±0.20^A^ | 7.62±0.58^BC^ | - | 100.00±0.00^A^ | - | 4.64±0.27^G^ |
| BH-959 | 0.71±0.07^A^ | 7.03±1.09^C^ | - | 100.00±0.00^A^ | - | 4.10±0.29^G^ |

**Table S4.** Energy Dispersive X-Ray Analyzer (EDX) of HPW-147gliadin, MCTLG-2 secalin and BH-393 hordein fractions

Mean±SD with different superscripts in column differ significantly (*p* ≤ 0.05); *n* = 3 for each treatment.

| **Element** | **Gliadin** | **Secalin** | **Hordein** | **Gliadin** | **Secalin** | **Hordein** |
| --- | --- | --- | --- | --- | --- | --- |
|  | **Atomic%** | | | **Weight%** | | |
| **C** | 57.60±3.00^A^ | 55.68±4.53^A^ | 52.33±203^A^ | 48.59±3.66^A^ | 50.71±4.44^A^ | 43.16±1.86^A^ |
| **N** | 13.79±2.77^A^ | 14.20±2.01^A^ | 10.43±1.51^A^ | 14.80±3.38^A^ | 9.46±4.92^A^ | 10.63±1.78^A^ |
| **O** | 27.03±1.18^B^ | 29.36±2.79^AB^ | 35.26±2.94^A^ | 30.49±1.89^B^ | 34.99±0.74^AB^ | 38.94±3.14^A^ |
| **Na** | 0.51±0.11^B^ | 0.28±0.08^C^ | 0.75±0.07^A^ | 0.83±0.17^B^ | 0.42±0.13^B^ | 1.18±0.11^A^ |
| **P** | 0.37±0.02^B^ | - | 0.61±0.09^A^ | 0.84±0.04^B^ | - | 1.31±0.19^A^ |
| **S** | 0.26±0.01^A^ | 0.23±0.10^A^ | 0.12±0.04^A^ | 0.63±0.02^A^ | 0.46±0.11^A^ | 0.25±0.09^B^ |
| **I** | 0.43±0.19^A^ | 0.25±0.10^A^ | 0.54±0.03^A^ | 3.83±1.60^A^ | 1.97±1.39^A^ | 4.56±0.26^A^ |

**Table S5.** Crystallite size (nm) of protein against Bragg’s angle (2θ, equation 5) showing crystalline structure of gliadin, secalin and hordein fractions by X-ray diffraction technique.

| **Gliadin** | **Peak Position (2θ)** | **Crystallite size (nm)** | **Secalin** | **Peak Position (2θ)** | **Crystallite size (nm)** | **Hordein** | **Peak Position (2θ)** | **Crystallite size (nm)** |
| --- | --- | --- | --- | --- | --- | --- | --- | --- |
| **HPW-42** | 44.06 | 12.66 | **MCTLG-1** | 44.08 | 12.66 | **BH-393** | 44.04 | 12.29 |
|  | - | - |  | 37.83 | 14.84 |  | 37.81 | 14.08 |
|  | 10.43 | 17.94 |  | 10.41 | 16.79 |  | 10.41 | 17.16 |
| **HPW-147** | 44.06 | 13.54 | **MCTLG-2** | 44.06 | 12.47 | **BH-902** | 44.07 | 13.05 |
|  | 37.83 | 11.23 |  | 37.86 | 15.68 |  | 37.82 | 16.63 |
|  | 10.41 | 13.05 |  | 10.41 | 15.47 |  | 10.38 | 17.94 |
| **HPW-155** | 44.04 | 12.29 | **MCTLG-3** | 44.08 | 12.66 | **BH-946** | 44.09 | 14.13 |
|  | - | - |  | 37.83 | 14.33 |  | - | - |
|  | 10.43 | 16.11 |  | 10.38 | 17.54 |  | 10.39 | 16.79 |
| **HPW-236** | 44.11 | 13.67 | **MCTLG-4** | 44.07 | 13.05 | **BH-959** | 44.05 | 12.47 |
|  | 37.87 | 14.33 |  | 37.92 | 14.01 |  | 37.78 | 13.4 |
|  | 10.42 | 17.94 |  | 10.41 | 16.79 |  | 10.40 | 17.54 |
| **HPW-249** | 44.11 | 12.47 | **MCTLG-5** | 44.09 | 12.85 |  |  | |
|  | - | - |  | 37.77 | 17.31 |  |  |  |
|  | 10.40 | 17.61 |  | 10.41 | 18.35 |  |  |  |
| **HPW-349** | 44.11 | 12.29 |  | | |  |  |  |
|  | 37.82 | 13.62 |  |  |  |  |  |  |
|  | 10.41 | 15.78 |  |  |  |  |  |  |

**Table S6.** Comparative d-spacing of protein showing crystalline structure by X-ray diffraction (against Bragg’s angle 2θ) and TEM SAED (Ring and Lattice diffraction) technique.

| **Gliadin** | **d-Spacing (nm)** | | | **Secalin** | **d-Spacing (nm)** | | | **Hordein** | **d-Spacing (nm)** | | |
| --- | --- | --- | --- | --- | --- | --- | --- | --- | --- | --- | --- |
|  | **XRD** | **TEM SAED Lattice diffraction** | **TEM SAED Ring diffraction** |  | **XRD** | **TEM SAED Lattice diffraction** | **TEM SAED Ring diffraction** |  | **XRD** | **TEM SAED Lattice diffraction** | **TEM SAED Ring diffraction** |
| HPW-42 | 0.21 | 0.29±0.65 | 0.21 | MCTLG-1 | 0.21 | - | - | BH-393 | 0.21 | 0.35±0.07 | 0.32 |
|  | 0.85 | - | - |  | 0.24 | - | - |  | 0.24 | - | - |
| HPW-147 | 0.21 | - | - |  | 0.85 | - | - |  | 0.85 | - | - |
|  | 0.24 | - | - | MCTLG-2 | 0.21 | - | - | BH-902 | 0.21 | - | - |
|  | 0.85 | - | - |  | 0.24 | - | - |  | 0.24 | - | - |
| HPW-155 | 0.21 | - | - |  | 0.85 | - | - |  | 0.85 | - | - |
|  | 0.85 | - | - | MCTLG-3 | 0.21 | - | - | BH-946 | 0.21 | - | - |
| HPW-236 | 0.21 | - | - |  | 0.24 | - | - |  | - | - | - |
|  | 0.24 | - | - |  | 0.85 | - | - |  | 0.85 | - | - |
|  | 0.85 | - | - | MCTLG-4 | 0.21 | - | - | BH-959 | 0.21 | - | - |
| HPW-249 | 0.21 | - | - |  | 0.24 | - | - |  | 0.24 | - | - |
|  | 0.85 | - | - |  | 0.85 | - | - |  | 0.85 | - | - |
| HPW-349 | 0.21 | - | - | MCTLG-5 | 0.21 | 0.32±0.14 | 0.21 |  |  | | |
|  | 0.24 | - | - |  | 0.24 | - | 0.34 |  |  |  |  |
|  | 0.85 | - | - |  | 0.85 | - | - |  |  |  |  |


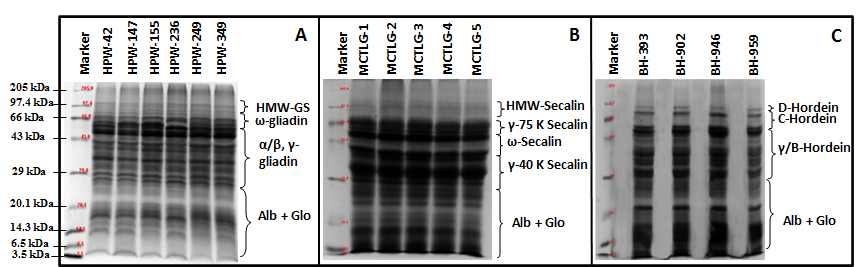


**Figure S1.** SDS-PAGE of (A) Gliadin (B) Secalin (C) Hordein extracted from different cultivars of wheat, rye and barley under reduced condition**.**


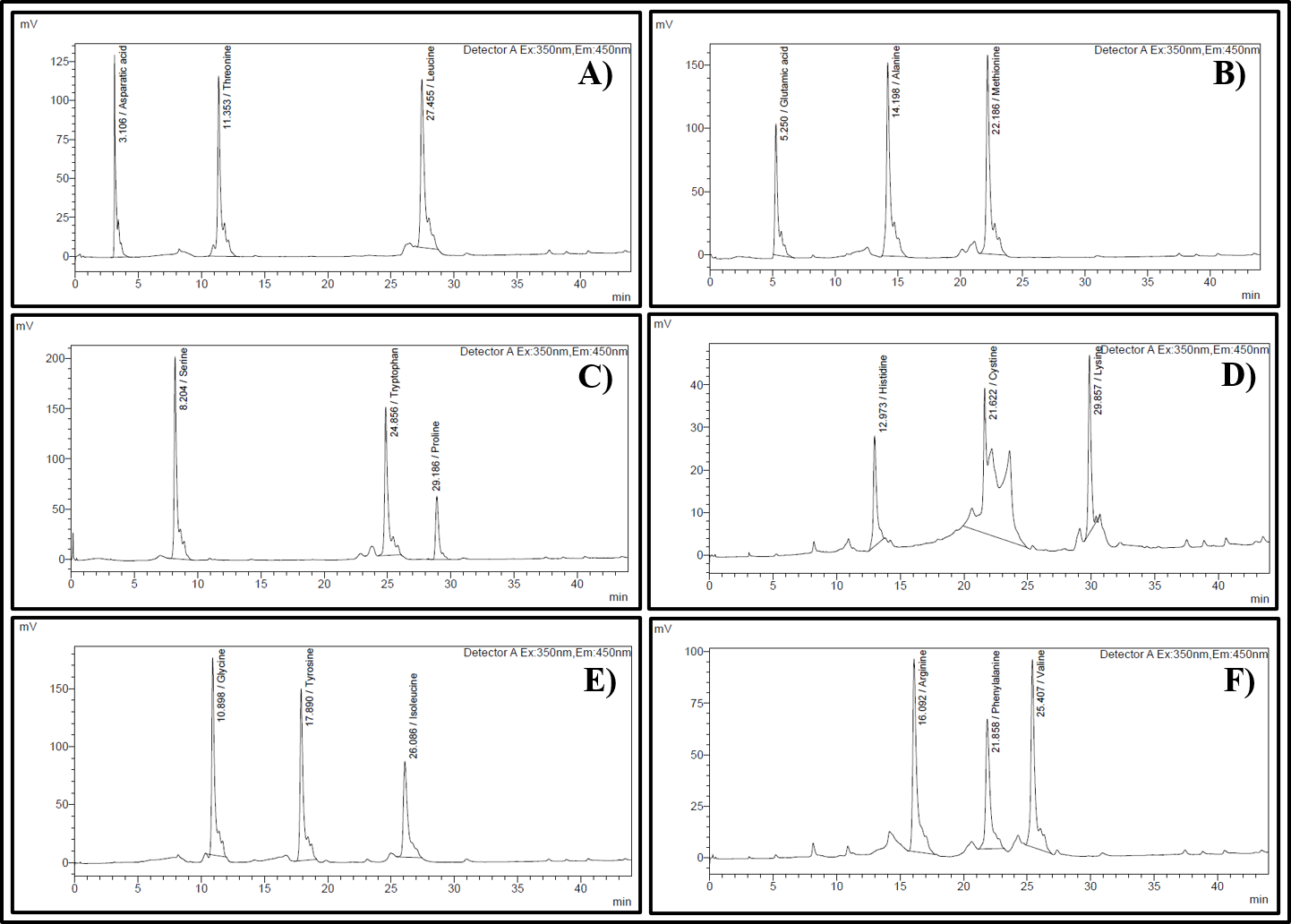


**Figure S2.** Peak identification by eluting three pair set of 18 standard amino acids to confirm the elution of amino acid series in the standard: (A) Asparagine+Aspartic acid-Threonine-Leucine, (B) Glutamine+Glutamic acid-Alanine-Methionine, (C) serine-Tryptophane-Proline, (D) Histidine-Cysteine-Lysine, (E) Glycine-Tyrosine-Isoleucine (F) Arginine-Phenylalanine-Valine.


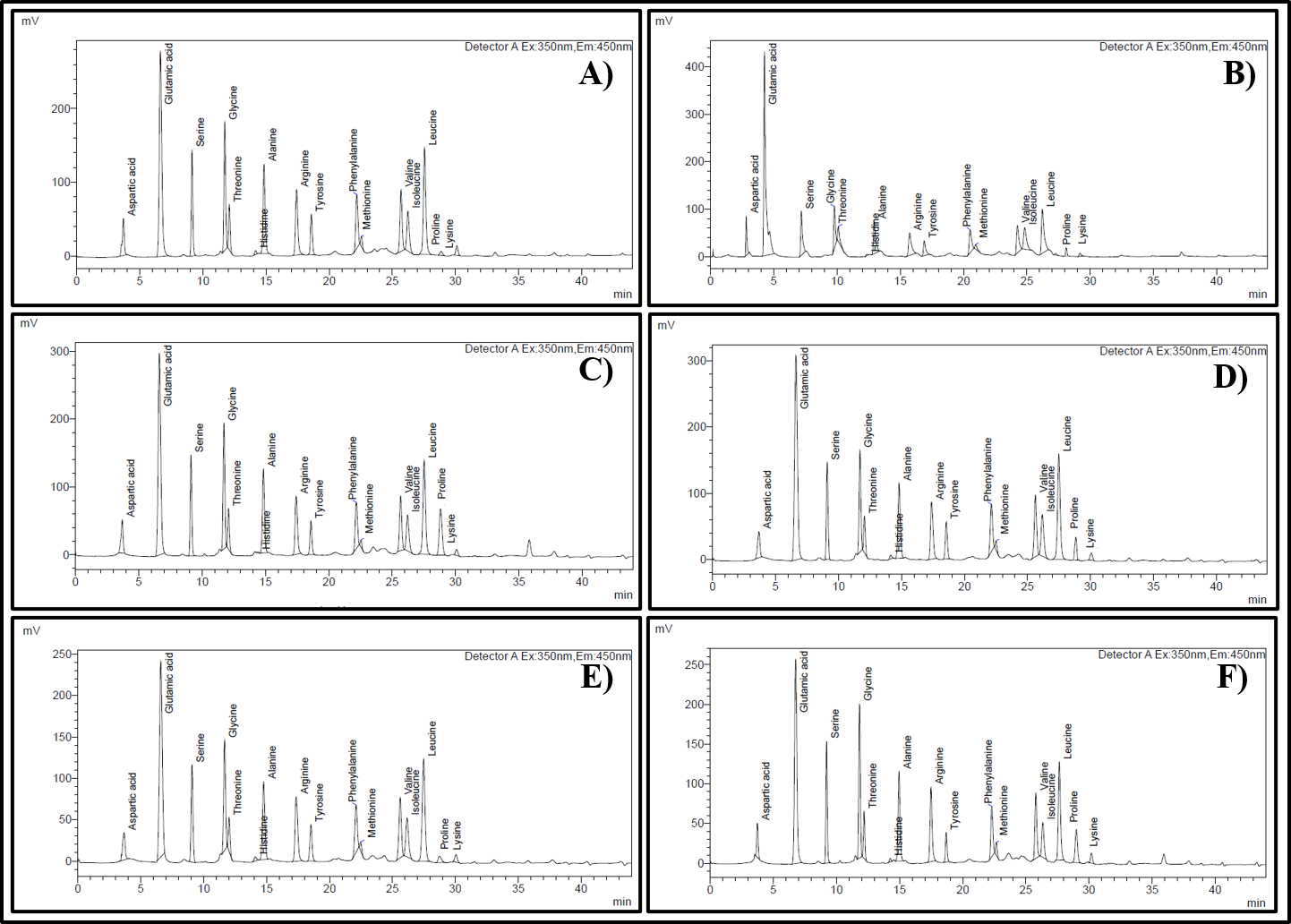


**Figure S3a.** HPLC Chromatograms displaying gliadin fraction A) HPW-42, B) HPW-155, C) HPW-236, D) HPW-249, E) HPW-349; secalin F) MCTLG-1.


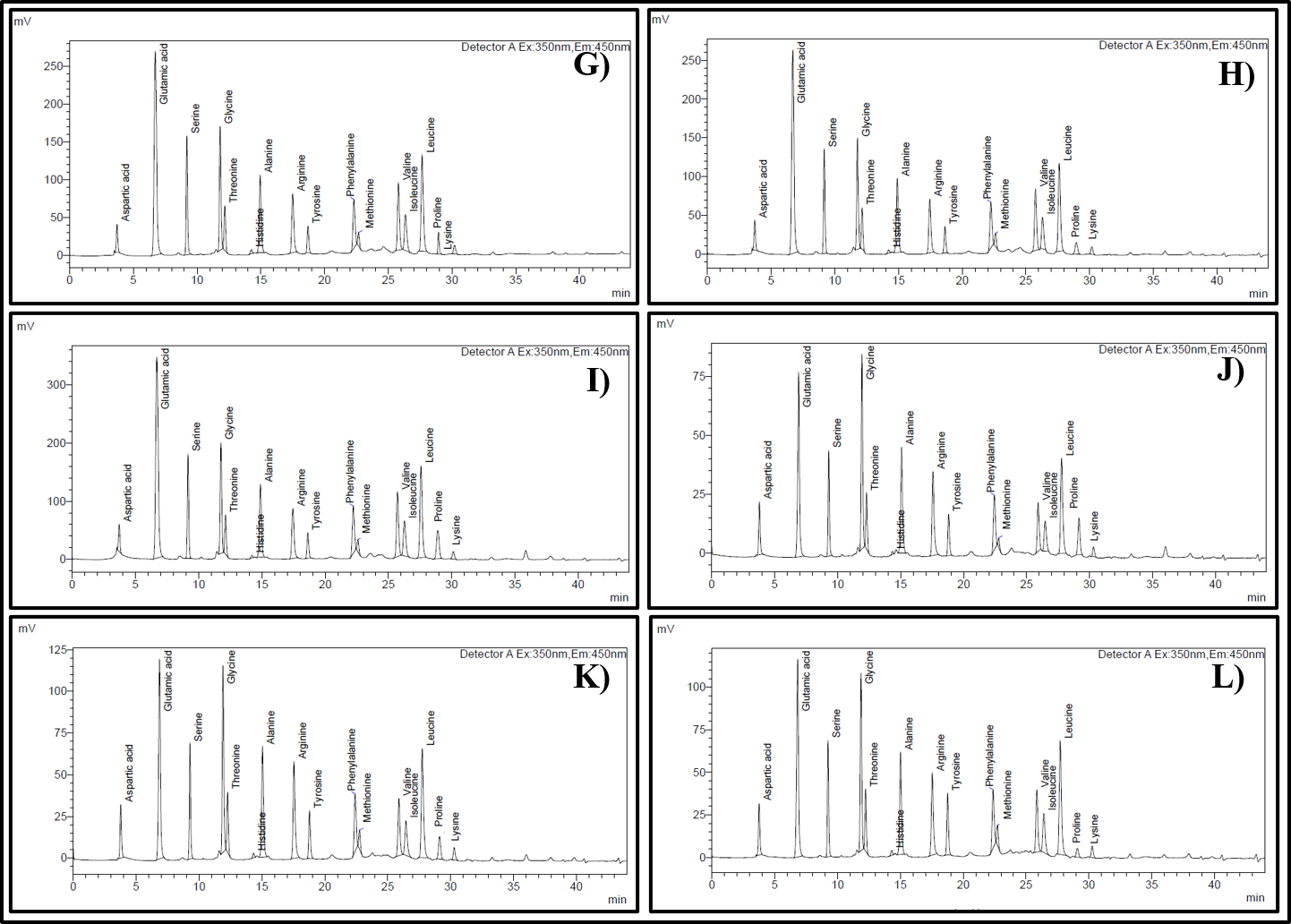


**Figure S3b.** HPLC Chromatograms displaying Amino acid profiling of secalin fraction G) MCTLG-3, H) MCTLG-4, I) MCTLG-5; Hordein J) BH-902, K) BH-946 L) BH-959.


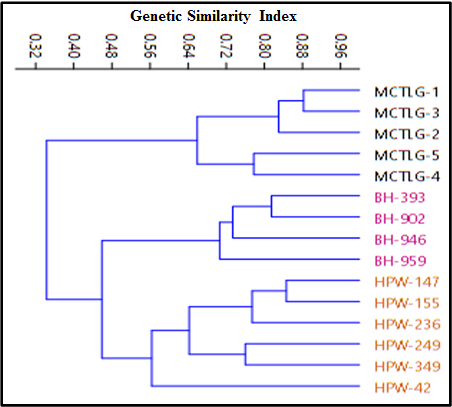


**Figure S4.** The dendrogram of wheat, rye and barley cultivars based on protein bands on SDS-PAGE using Jaccard Similarity Coefficient and Un-weighted Paired Group Method with Arithmetic Mean (UPGMA).

**Figure S5a.** Curve fitting and statistical analysis of FTIR spectrum showing amide I (1600–1700) region of Gliadin fraction A) HPW-147, B) HPW-155, C) HPW-236, D) HPW-249, E) HPW-349; Hordein fraction F) BH-902.


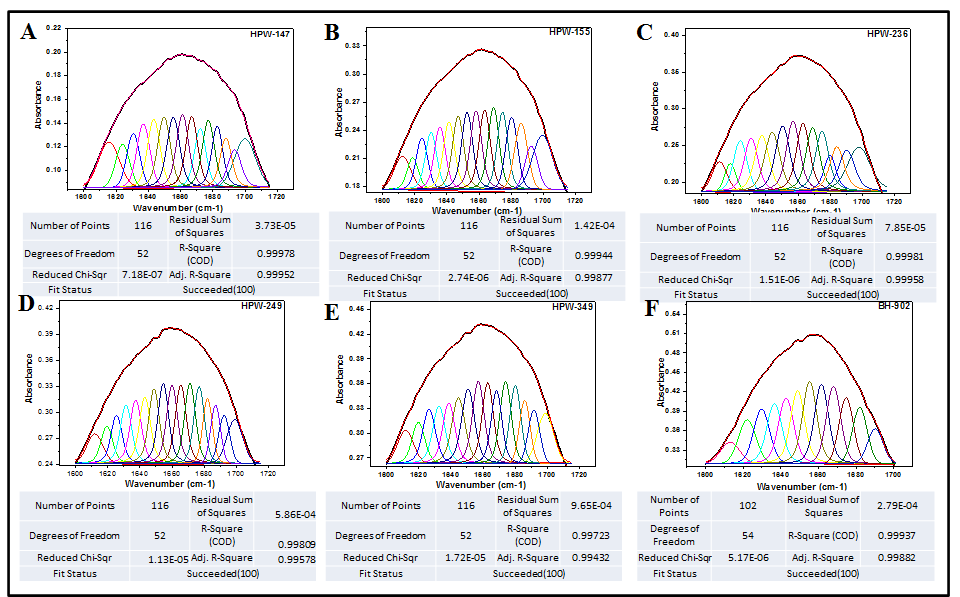


**Figure S5b.** Curve fitting and statistical analysis of FTIR spectrum showing amide I (1600–1700) region of Hordein fraction G) BH-946, H) BH-959; Secalin fraction I) MCTLG-1, J) MCTLG-2, K) MCTLG-3 and L) MCTLG-4.


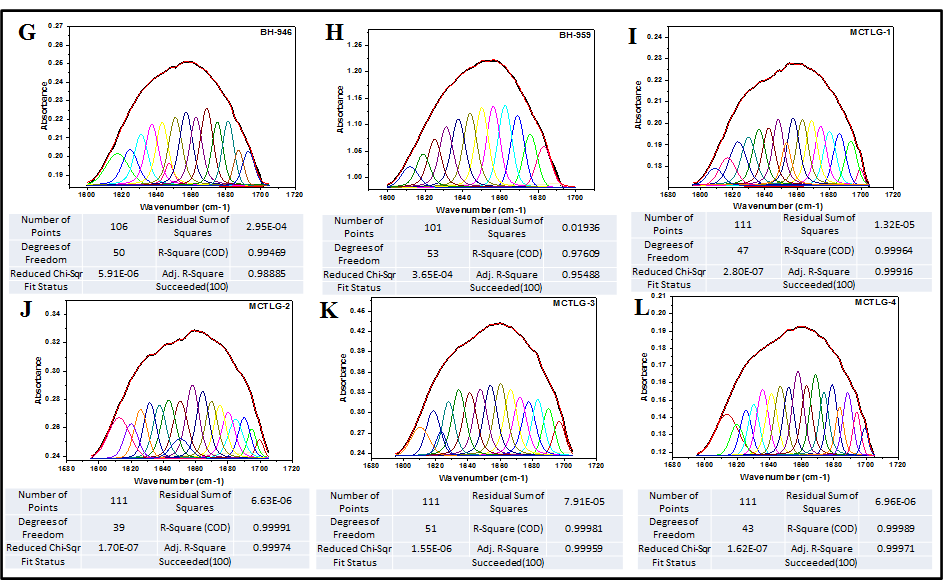


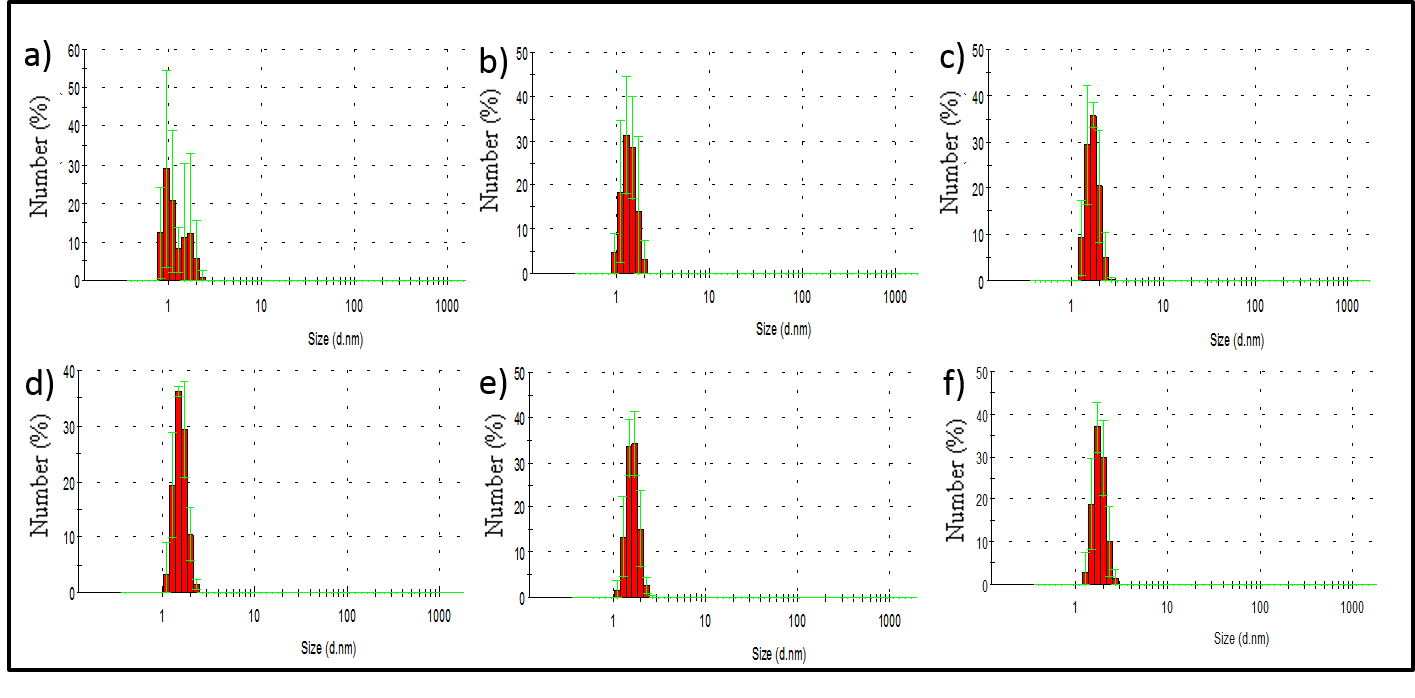
**Figure S6a.** DLS images showing number-based size distribution of Gliadin fraction a) HPW-42, b) HPW-147, c) HPW-155, d) HPW-236, e) HPW-249 and f) HPW-349.


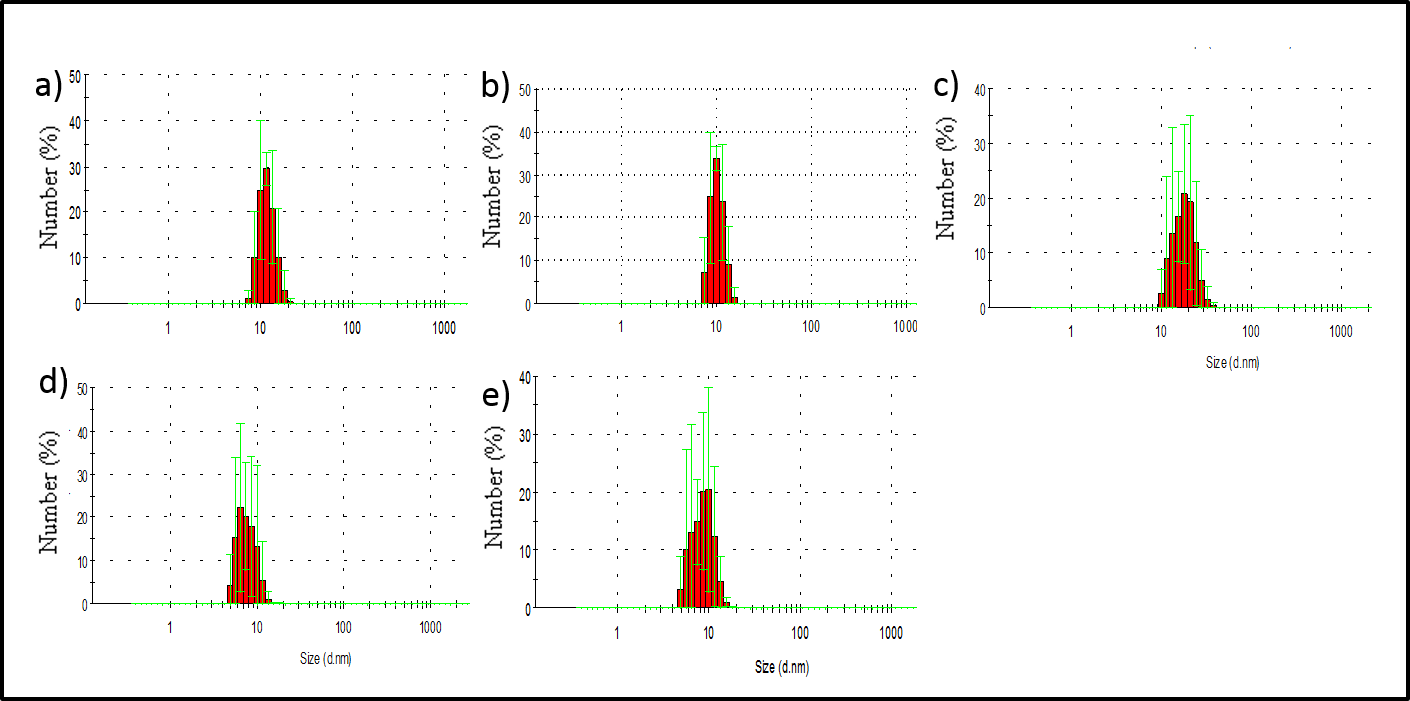


**Figure S6b.** DLS images showing number-based size distribution of secalin fraction a) MCTLG-1, b) MCTLG-2, c) MCTLG-3, d) MCTLG-4, and e) MCTLG-5.


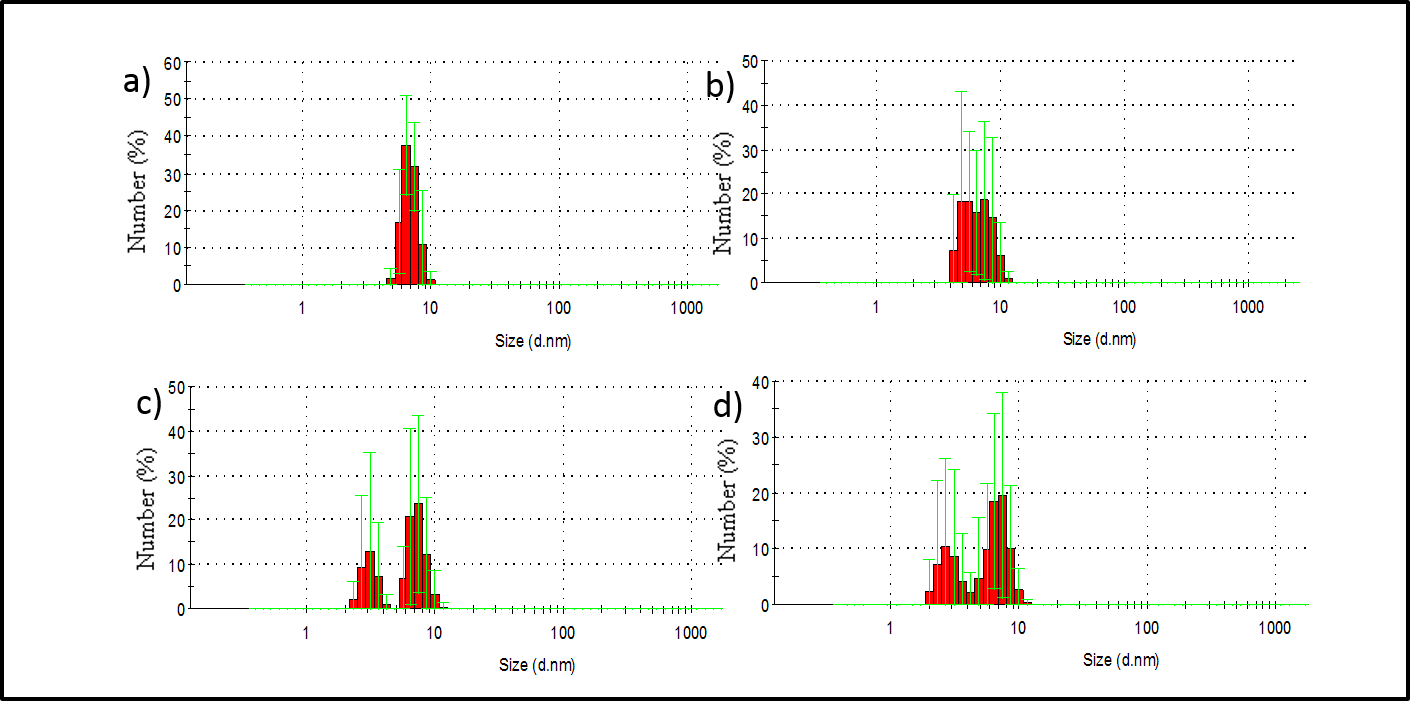


**Figure S6c.** DLS images showing number-based size distribution of hordein fraction a) BH-393, b) BH-902, c) BH-946 and d) BH-959 extracted with DuPont extraction protocol.


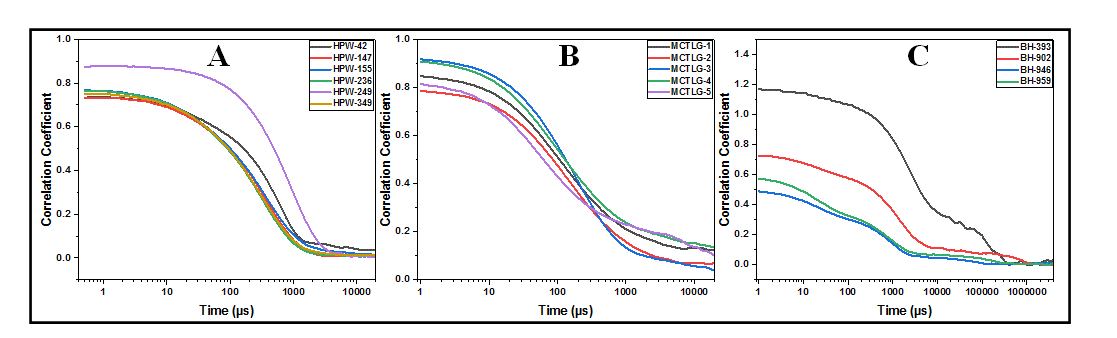


Figure S6d. The correlogram of A) Gliadin, B) Secalin and C) Hordein obtained corresponding to their respective cultivars.


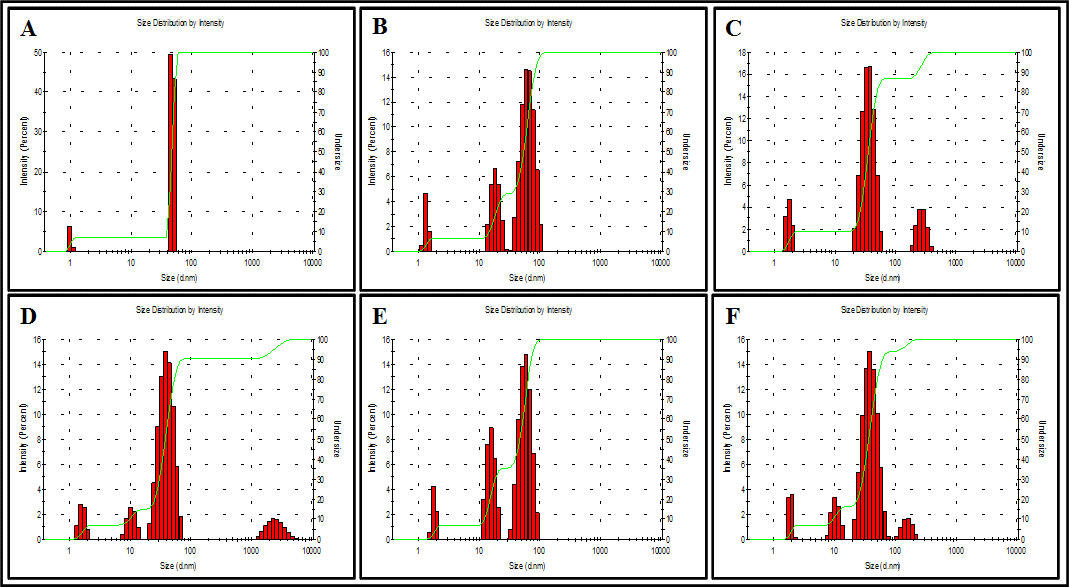


**Figure S6e.** DLS images showing intensity-based size distribution of Gliadin fraction a) HPW-42, b) HPW-147, c) HPW-155, d) HPW-236, e) HPW-249 and f) HPW-349.


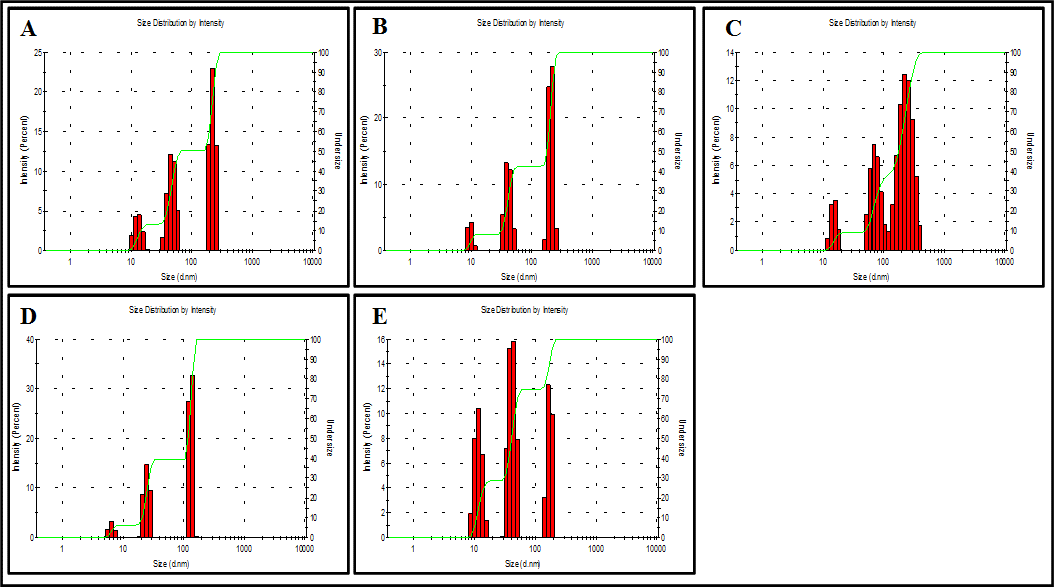


**Figure S6f.** DLS images showing intensity-based size distribution of secalin fraction a) MCTLG-1, b) MCTLG-2, c) MCTLG-3, d) MCTLG-4, and e) MCTLG-5.


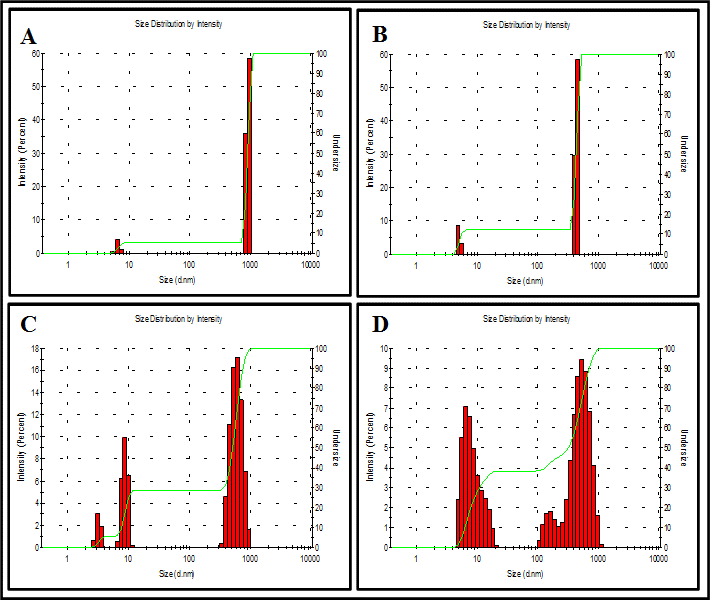


**Figure S6g.** DLS images showing intensity-based size distribution of hordein fraction a) BH-393, b) BH-902, c) BH-946 and d) BH-959 extracted with DuPont extraction protocol.


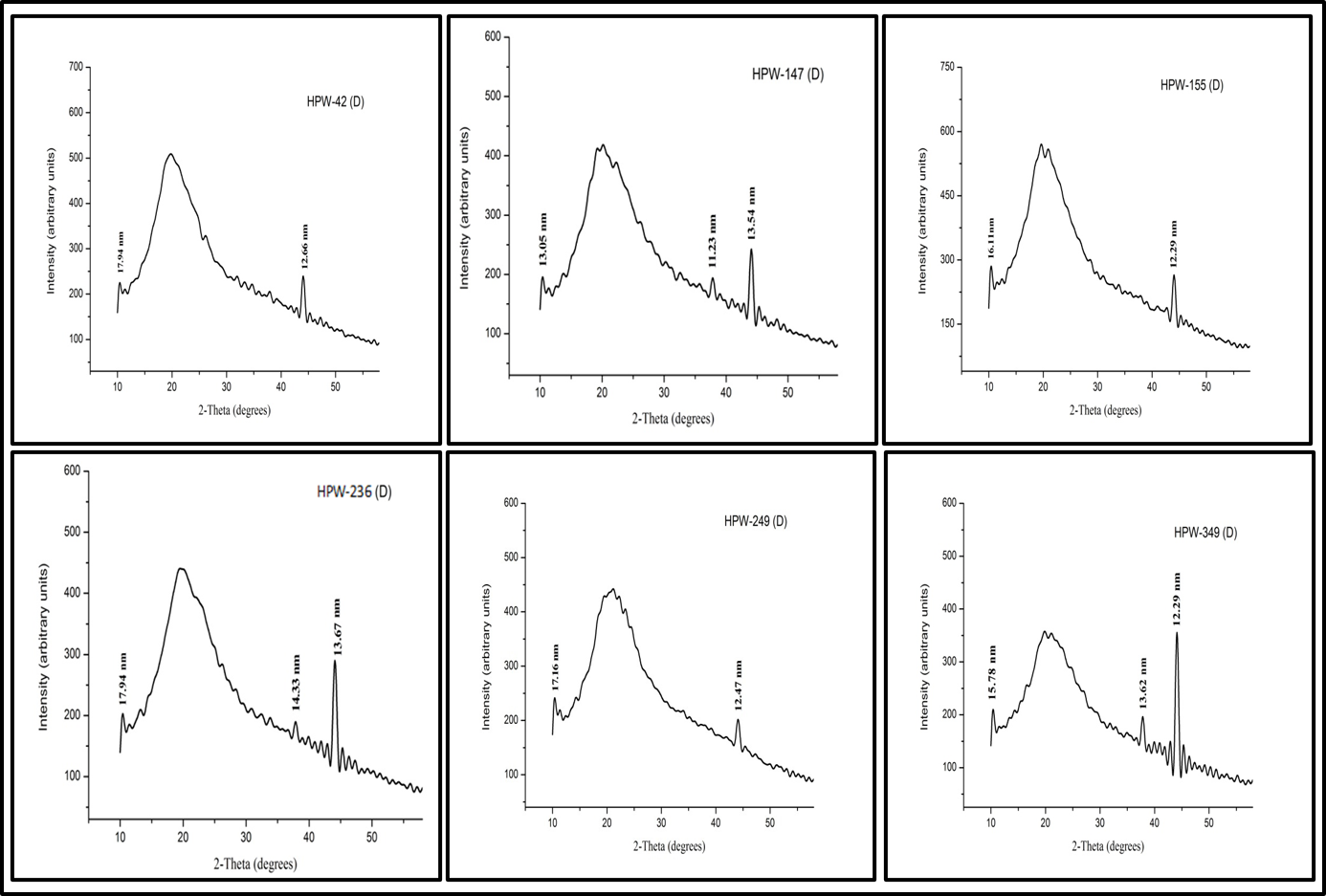


**Figure S7a.**X-ray diffractograms smoothened through 1 Hz low pass FFT filters of gliadin fraction.


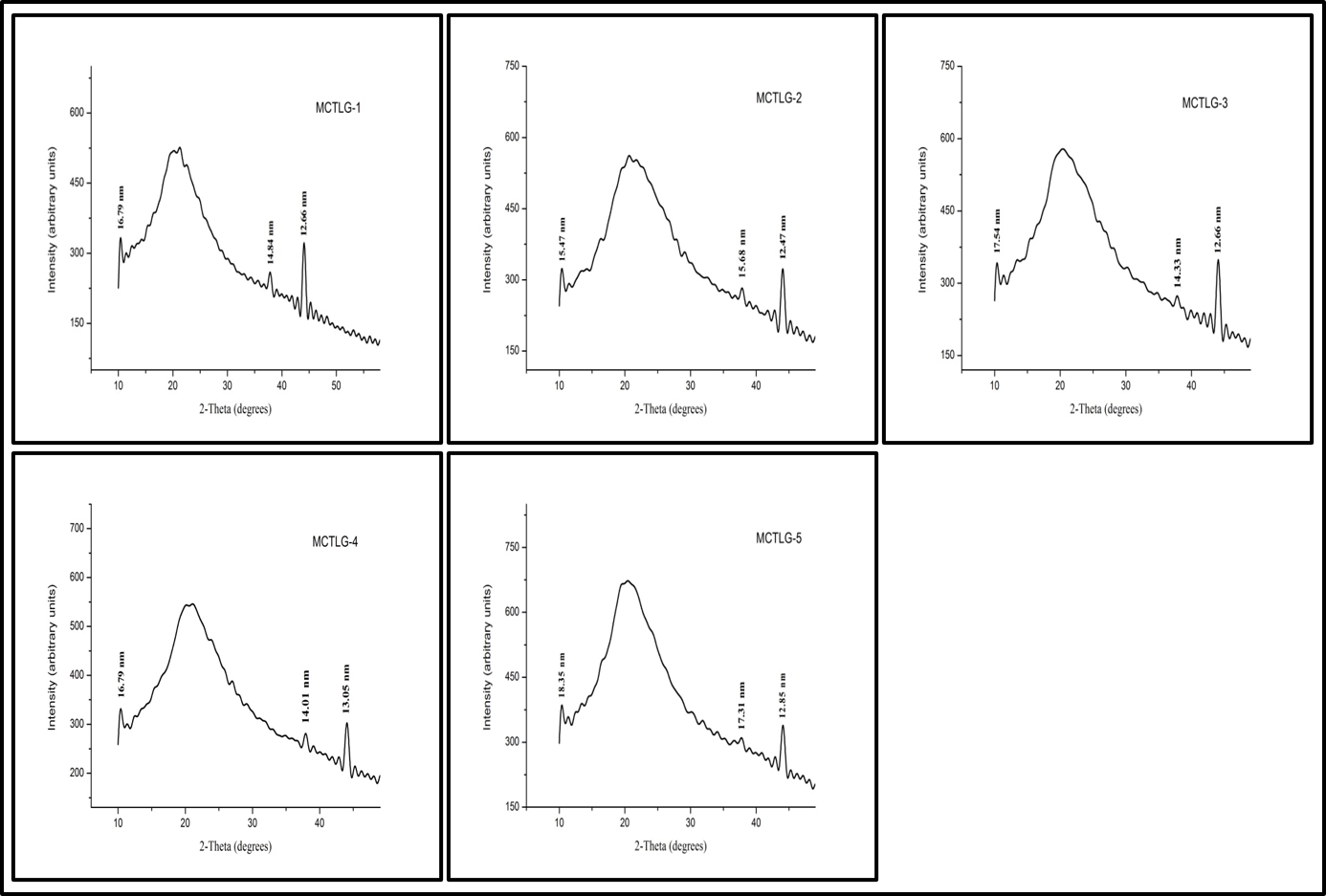


**Figure S7b.**X-ray diffractograms smoothened through 1 Hz low pass FFT filters of secalin fraction


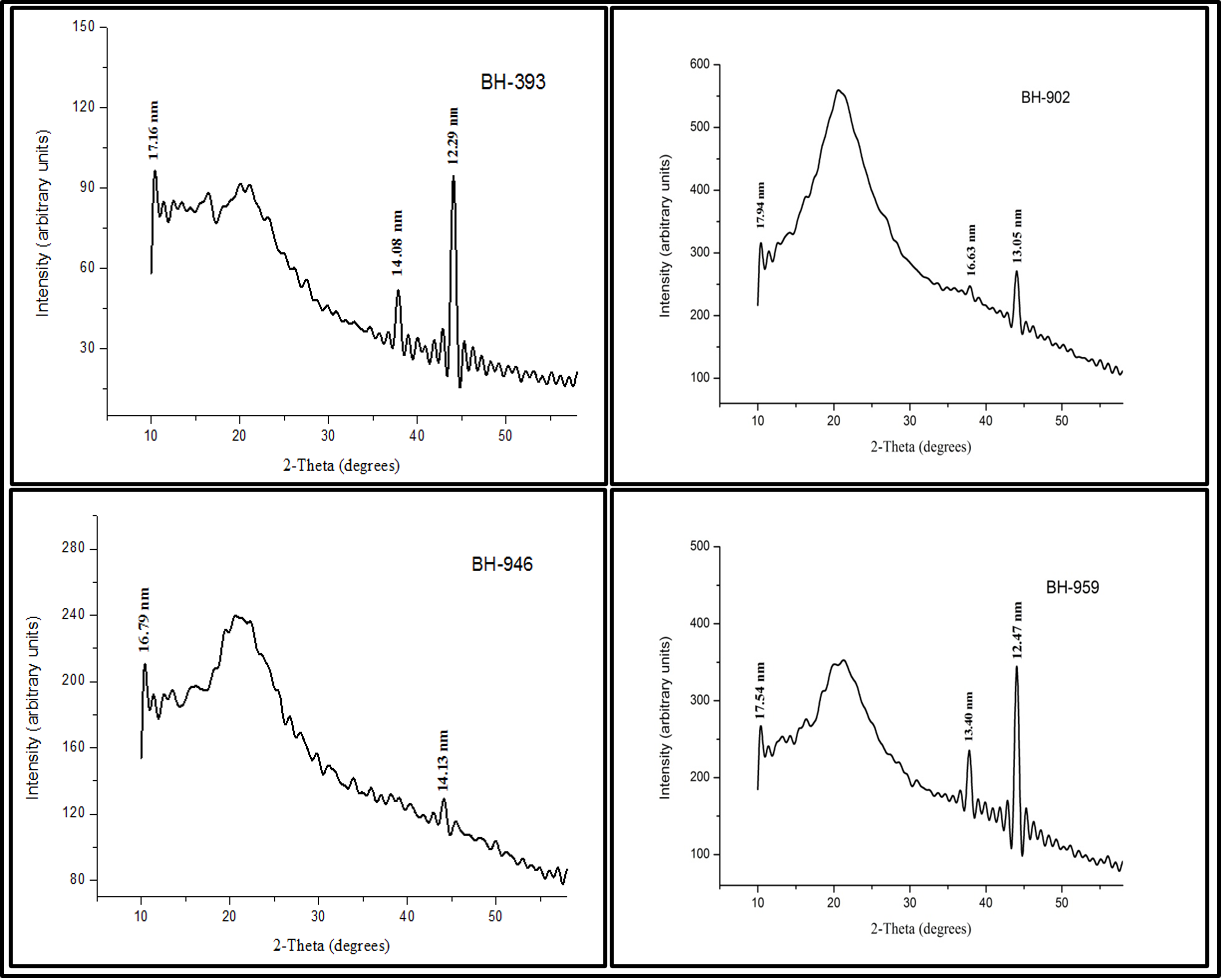


**Figure S7c.**X-ray diffractograms smoothened through 1 Hz low pass FFT filters of hordein fraction.


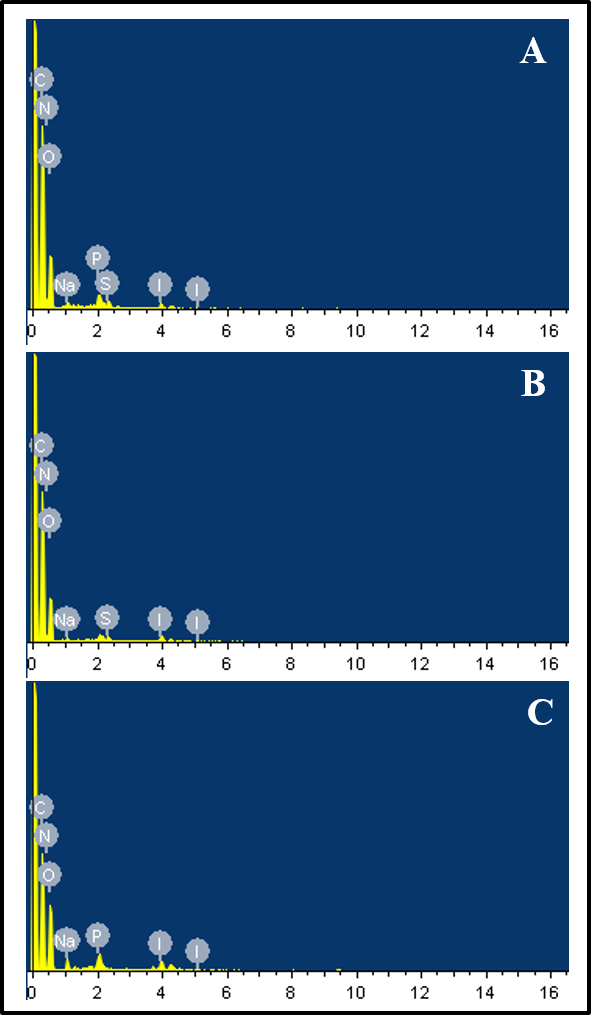


**Figure S8.** Energy Dispersive X-Ray Analyzer (EDX) spectra of (A) HPW-42 gliadin, (B) MCTLG-5 secalin and (C) BH-393 hordein fractions.


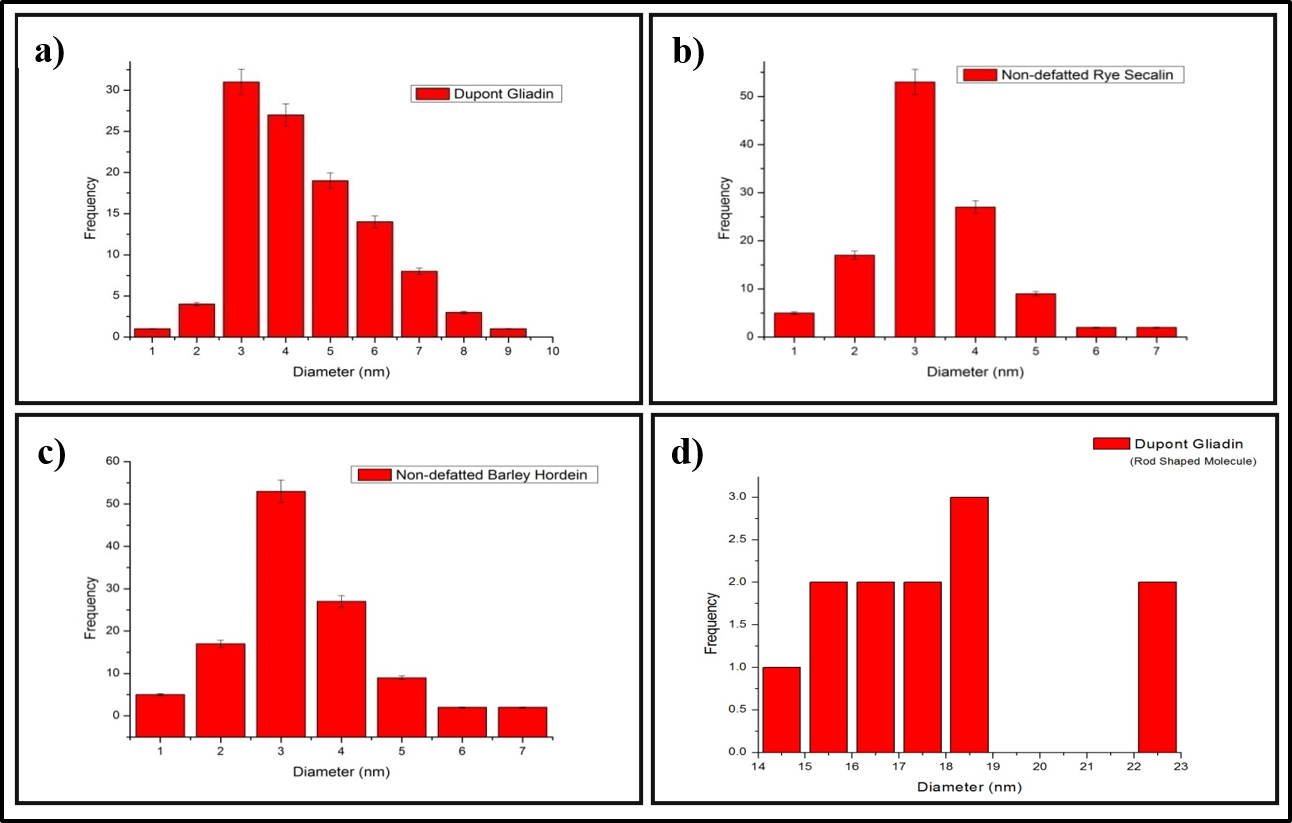


**Figure S9.** Size of prolamin fraction from TEM Images (a) Gliadin (b) Secalin (c) Hordein and d) Gliadin (rod shaped peptides).
